# Rethinking exercise dose for cognitive ageing: a systematic review and meta-analysis of physical dose and cognitive–motor demands

**DOI:** 10.3389/fpsyg.2026.1906526

**Published:** 2026-08-13

**Authors:** Daniela De Bartolo, Liliana Baleca, Claudia Salera, Fabio Lucidi

**Affiliations:** 1 Department of Psychology of Developmental and Socialisation Processes, Faculty of Medicine and Psychology, Sapienza University of Rome, Rome, Italy; 2 SmArt Lab—Laboratory for the Study of Mind and Action in Rehabilitation Technologies, IRCCS Foundation Santa Lucia, Rome, Italy; 3 Department of Psychology, Faculty of Medicine and Psychology, Sapienza University of Rome, Rome, Italy

**Keywords:** cognitive ageing, exercise dose, healthy ageing, randomized controlled trials, structured exercise

## Abstract

Exercise is widely promoted for cognitive ageing, yet the field still lacks a clear account of what constitutes an effective “dose” when cognition is the target. From a movement-science perspective, this distinction is important because exercise interventions may differ not only in physical exposure, but also in the perceptual, attentional, coordinative and adaptive demands embedded in movement. Most trials define dose through physical exposure variables, including frequency, duration, weekly volume, intervention length and intensity. However, these metrics do not capture what the exercise task requires cognitively or motorically. We conducted a systematic review and meta-analysis of randomized controlled trials testing structured exercise in community-dwelling older adults without dementia. Cognitive outcomes were grouped into global cognition and domain-specific outcomes. Hedges’ g was calculated from post-intervention means and standard deviations, with positive values favoring exercise. Random-effects and multilevel models estimated pooled effects and tested whether weekly volume, intervention duration and a structured cognitive–motor demand score explained between-study variability. Thirty-seven trials provided 166 effect-size estimates. Structured exercise was associated with better global cognition (*g* = 0.55, 95% CI 0.38 to 0.73), although heterogeneity was substantial and certainty was very low. Domain-specific effects were smaller and generally favorable, but none remained significant after false discovery rate correction. Weekly volume and intervention duration did not consistently explain cognitive effects. Cognitive–motor demand showed only partial overlap with physical-dose indicators, suggesting that it captured a distinct qualitative dimension of exercise content; however, it did not explain effects beyond physical dose. Component-level analyses suggested possible domain-specific patterns for set shifting. Current trials are therefore not yet able to define a precise cognitive dose of exercise. Future studies should test physical dose and cognitive–motor task demands as distinct, potentially interacting dimensions of exercise for cognitive ageing.

**Systematic review registration:**

https://www.crd.york.ac.uk/PROSPERO/view/CRD420250622607.

## 1 Introduction

With population ageing, safeguarding cognitive function is becoming an increasingly important aspect of healthy and active ageing. Even in the absence of neurological disorders, ageing is commonly associated with slower processing, reduced working memory, weaker executive control, lower cognitive flexibility and poorer set shifting (Salthouse, 2009; Harada et al., 2013; Murman, 2015). These cognitive functions support everyday behaviors such as decision-making, planning, adaptation to new situations and independent living. Their decline may therefore increase vulnerability to subsequent cognitive impairment and dementia (Harada et al., 2013; Diamond, 2013; Livingston et al., 2017).

Aerobic exercise is one of the most studied non-pharmacological strategies for preserving cognitive function across the lifespan (Voss et al., 2011). Previous reviews have examined the effects of physical activity and structured exercise on global cognition and specific cognitive domains, and several randomized trials have reported benefits for global cognition, executive function and processing speed (Colcombe and Kramer, 2003; Northey et al., 2018; Chen et al., 2020). These effects are biologically plausible because exercise may influence cerebrovascular function, neurotrophic signalling and plasticity in brain regions involved in cognition, including the hippocampus and prefrontal cortex (Erickson et al., 2011; Voss et al., 2011; Szuhany et al., 2015; Stillman et al., 2020; Bliss et al., 2021). However, translating this evidence into cognitive-oriented exercise prescription remains difficult. The key uncertainty is no longer only whether exercise can support cognition, but also which characteristics of exercise should be prioritised when cognitive outcomes are the target. This perspective is particularly relevant for movement science, where exercise can be considered not only as a physiological exposure, but also as a structured motor behavior requiring perception, action selection, coordination, monitoring and adaptation.

In exercise-cognition research, dose has most often been conceptualized through the same variables used in general exercise prescription. Exercise guidelines typically define dose using physical exposure metrics such as session duration, weekly frequency, intervention length, total minutes and physiological intensity (Garber et al., 2011; van der Ploeg and Bull, 2020). This approach is clinically useful because these parameters are measurable, prescribable and directly relevant for safety, feasibility and physiological adaptation.

Two influential meta-analyses illustrate both what is known about exercise dose and what remains unresolved. Northey et al. (2018) examined exercise modality, session duration, frequency, physiological intensity and intervention length in adults older than 50 years. Exercise was associated with better cognitive performance overall, with favourable estimates for interventions involving 45–60-min sessions and at least moderate intensity. However, confidence intervals across moderator categories overlapped substantially, and most trials were concentrated around similar exercise prescriptions. Sanders et al. (2019) examined programme duration, session duration, frequency, intensity and cumulative exercise exposure, defined from the combination of programme length, session duration and frequency. Among cognitively healthy older adults, exercise showed small positive effects on executive function and memory, but none of the investigated dose parameters predicted the magnitude of these effects. Associations with shorter sessions and higher frequency were observed only among participants with cognitive impairment.

These findings indicate that conventional dose variables are important for describing exercise exposure but have not yielded a consistent prescription for cognitive benefit. Their interpretation is limited by the restricted variation in dose across trials, incomplete reporting of intensity and adherence, and the fact that most studies did not experimentally compare different doses within the same intervention. Moreover, these variables quantify how much exercise was prescribed without describing the cognitive and motor operations required to perform it. The present review therefore updated the randomized evidence in community-dwelling older adults without dementia and examined physical-dose indicators at the intervention-arm level using linear, categorical and exploratory non-linear models. It additionally evaluated whether cognitive–motor task demands could be coded separately from physical exposure and whether they explained variability in cognitive outcomes beyond conventional dose indicators.

The available evidence remains heterogeneous and sometimes inconclusive, suggesting that simply increasing exercise volume does not necessarily produce proportionally larger cognitive benefits (Northey et al., 2018; Sanders et al., 2019). This uncertainty limits the ability to determine not only whether exercise supports cognition, but also how exercise should be prescribed to optimize cognitive outcomes. More importantly, it raises a conceptual problem: physical-dose models describe how much exercise was delivered, but they do not necessarily describe what kind of cognitive and motor demands participants experienced during the intervention. This concern is consistent with previous calls to shift the focus in exercise–cognition research from purely quantitative exercise characteristics to qualitative features of movement and task engagement (Garber et al., 2011; Pesce, 2012).

A dose model based only on physical exposure may be too narrow for cognitive ageing research because exercise is not unitary exposure. Activities with similar weekly volume or physiological intensity may differ substantially in their cognitive–motor demands. Continuous walking or cycling, resistance training, dance, Tai Chi, martial arts, exergaming and multicomponent exercise may all involve physical effort, but they differ in movement variability, coordination, sequencing, balance control, attentional engagement, decision-making, social interaction and adaptability. Conversely, interventions with similar cognitive–motor demands may differ in duration, weekly volume or physiological intensity. These qualitative differences may influence the cognitive processes engaged during training, because participants may be required to perceive, monitor, select, inhibit, remember and adapt actions while moving. Different forms of physical activity may also be associated with partly distinct structural and functional brain adaptations, especially when metabolic and coordinative demands are considered separately (Voelcker-Rehage and Niemann, 2013).

The distinction between physical exposure and cognitive–motor task demands is supported by theoretical and empirical work. Evidence from motor-cognitive training suggests that cognitive and motor components may interact differently depending on whether they are trained separately, concurrently or in an integrated manner (Herold et al., 2019). Accordingly, previous work has argued for greater attention to qualitative features of movement, including motor complexity and cognitive engagement (Pesce, 2012; Gu et al., 2019). Evidence from motor-cognitive interdependence, experience-dependent plasticity, open-skill exercise and combined motor–cognitive training further supports the idea that the cognitive effects of exercise may depend on more than physical volume alone (Schäfer et al., 2006; Schneider and Yvon, 2013; Gu et al., 2019). Despite this rationale, randomized trials and meta-analyses often classify interventions using broad modality labels such as aerobics, resistance, multicomponent or mind–body training. These categories are useful for summarizing the literature, but they may obscure the intervention features that are most relevant to cognition. For example, two interventions classified as aerobic exercise may differ markedly in coordination and decision-making demands, whereas a mind–body or dance intervention may combine moderate physiological intensity with high cognitive–motor engagement. Conversely, interventions with similar cognitive–motor demands may differ in intensity, duration or weekly volume. Distinguishing physical dose from cognitive–motor demands may therefore help clarify why cognitive effects are modest, heterogeneous and difficult to translate into precise prescriptions.

The challenge is not only statistical, but conceptual. If cognitive change depends partly on the operations required by the task, then a cognitive dose of exercise cannot be inferred from minutes per week alone. It also requires information on the cognitive–motor structure of the intervention, including task variability, progression, cueing, balance demands, sequencing, decision-making and adaptation. However, current trial reports often provide more detail on physical prescription variables than on these task features. As a result, it remains difficult to determine whether cognitive outcomes are related to physical dose, to cognitive–motor task demands, or to their combination. The present systematic review and dose–response meta-analysis therefore aimed to synthesize randomized controlled trials of structured exercise interventions in older adults without dementia while evaluating how exercise dose is defined in this literature. We examined whether conventional physical-dose indicators, including weekly volume, intervention duration and reported intensity, were associated with variability in cognitive effects. We then explored whether cognitive–motor demands could be coded from published trial descriptions and whether this structured intervention-level classification showed preliminary associations with cognitive outcomes. Rather than treating exercise dose as a single quantitative exposure, we considered it as a multidimensional construct, distinguishing physical exposure from intervention-level cognitive–motor demands. By doing so, this review sought to clarify what current randomized trials can and cannot tell us about dose–response patterns in cognitive ageing. The aim was not only to estimate whether structured exercise is associated with cognitive benefit, but also to evaluate whether the existing evidence base is sufficiently designed and reported to define a precise cognitive dose of exercise.

## 2 Methods

### 2.1 Protocol and registration

This systematic review with meta-analysis was conducted and reported in accordance with the PRISMA 2020 statement (Page et al., 2021). The protocol was prospectively registered in PROSPERO (CRD420250622607) and updated as needed. The PRISMA flow diagram was generated using the PRISMA2020 R package and Shiny app (Haddaway et al., 2022).

The present review extends the original dose–response framework by explicitly distinguishing conventional physical-dose indicators from intervention-level cognitive–motor demands and by coding whether exercise arms included an additional cognitive stimulation component.

### 2.2 Search strategy and selection criteria

A systematic search was conducted in PubMed/MEDLINE, Scopus and Embase from inception to 5 February 2026. The search strategy combined controlled vocabulary (MeSH and Emtree) and free-text terms related to ageing, exercise and cognition. Searches were restricted to human randomized controlled trials published in peer-reviewed journals in English. Full search strategies are provided in Supplementary Table S1.

Eligibility criteria were defined using the PICOS framework (Spitzer et al., 1978). We included randomized controlled trials enrolling community-dwelling older adults without diagnosed dementia or major neurological or psychiatric disorders. Studies including participants with subjective cognitive complaints were eligible in the absence of a formal diagnosis of mild cognitive impairment or dementia.

Eligible interventions consisted of structured and repeated exercise programs delivered over multiple sessions. To maintain a focus on structured exercise prescription, we excluded interventions primarily designed as gait rehabilitation, walking rehabilitation or dual-task gait training, as these represent a partially distinct rehabilitation literature. Exercise interventions combined with cognitive stimulation or cognitive training were eligible when the experimental arm included a structured exercise component and the contrast allowed the added contribution of the exercise-containing intervention to be estimated. Because explicit cognitive stimulation could nevertheless influence cognitive outcomes independently of exercise, these arms were flagged *a priori* and excluded in sensitivity analyses. Cognitive training alone was not treated as an eligible experimental intervention but could serve as a comparator when the contrast estimated the additional effect of exercise or exercise combined with cognitive stimulation.

Comparators included passive controls, usual care, no-intervention conditions, active controls and, where appropriate, cognitive or educational control conditions. Studies were required to report objective cognitive outcomes assessed using standardized neuropsychological tests. Non-randomized studies, cross-over designs without extractable parallel-group data, conference abstracts, preprints and studies with insufficient post-intervention cognitive data were excluded.

### 2.3 Data extraction and quality assessment

Data was independently extracted by two reviewers (DDB and LB) using a standardized form. Extracted variables included study and participant characteristics, intervention and comparator details, cognitive outcomes, post-intervention means and standard deviations, sample sizes, intervention duration, weekly frequency, session duration, number of sessions, exercise intensity, modality, and information required to code cognitive–motor demands.

Cognitive outcomes were grouped according to established neuropsychological frameworks and previous exercise–cognition reviews (Colcombe and Kramer, 2003; Lezak, 2004; Northey et al., 2018; stillman et al., 2020). Domains included global cognition, executive functions, processing speed, set shifting, working memory, short-term memory, long-term memory, attention, selective attention, semantic fluency and phonemic fluency. Detailed classification procedures are reported in Supplementary Table S2.

A cognitive–motor demand index was developed by DDB, drawing on published frameworks concerning qualitative exercise characteristics and motor–cognitive training, and refined within the author team. Each intervention arm was independently coded by two reviewers (DDB and LB) for motor complexity and implicit cognitive demand, both scored from 0 to 4, and for the presence of explicit cognitive training, scored as 0 or 4. Component scores were summed to obtain a total score ranging from 0 to 12 and classified as low (0–2), moderate (3–5), or high (6–12). Eight predefined intervention profiles were used as coding anchors. Coding was conservative and based only on features explicitly reported in the original articles or Supplementary materials. Inter-rater agreement was calculated on the pre-consensus scores using quadratic-weighted Cohen’s kappa for the ordinal components and demand class, Cohen’s kappa for explicit cognitive training, and ICC (2,1) for the total score. Disagreements were resolved through discussion and re-examination of the source reports, with third-author adjudication planned if required.

Exercise interventions were categorized into seven modality groups based on predominant training characteristics, extending previous exercise–cognition classifications (Northey et al., 2018). Interventions combining exercise with other components were included when the exercise-containing contrast was interpretable for the review question; arms with explicit cognitive stimulation or cognitive training were coded separately and examined in sensitivity analyses.

Exercise intensity was classified as low, moderate or vigorous using established equivalence across %HR max, %VO₂ max, METs and Borg RPE scales (Garber et al., 2011). Details are provided in Supplementary Tables S3.1, S3.2. Conventional physical dose was coded using session duration, weekly frequency, intervention duration, number of sessions and weekly exercise volume. Weekly exercise volume was calculated as frequency per week multiplied by session duration and used as the primary physical-dose indicator; intervention duration in weeks was examined as a second dose dimension.

A structured cognitive–motor demand index was developed as an intervention-level proxy of qualitative task demands. It captured features reported in intervention descriptions, including movement variability, coordination, sequencing, balance and postural control, attentional engagement, decision-making, adaptability/progression and explicit cognitive–motor integration. The index was distinct from physiological intensity and from explicit cognitive stimulation. Explicit cognitive stimulation was coded separately as yes/no when the exercise arm included an added non-motor cognitive component, such as cognitive training, mental activity or computerized cognitive tasks. For descriptive analyses, interventions were grouped into low, moderate and high cognitive–motor demand classes. Because the index was retrospectively derived from published reports, it was used for exploratory evidence mapping and moderator analyses rather than confirmatory inference.

Risk of bias was assessed using the Cochrane Risk of Bias 2 tool (Sterne et al., 2019). Certainty of evidence for each outcome was evaluated using GRADE (Piggott et al., 2020).

### 2.4 Data synthesis

Hedges’ g was calculated as the standardised post-intervention difference between experimental and control groups. For outcomes where lower scores indicated better performance, effect sizes were reoriented so that positive values consistently favoured exercise. Random-effects meta-analyses were performed for each cognitive domain when at least three studies were available. To avoid within-domain duplication, each study contributed a single effect size per domain to the primary pooled models.

Primary pooled effects were estimated separately for each cognitive domain. Dose–response associations were examined using meta-regression models based on weekly exercise volume and intervention duration. Non-linear associations were explored using natural cubic splines with knots placed at prespecified quantiles of the observed dose distribution; given the limited and clustered distribution of weekly exercise volume, spline models were interpreted descriptively.

Moderator analyses used multilevel meta-regression models when multiple effect sizes per study were included, with random effects for study and robust variance estimation to account for dependence. Weekly exercise volume was also categorised into predefined bands (<90, 90–149, 150–224 and ≥225 min/week) to provide clinically interpretable exposure groups around common physical activity recommendations.

Additional exploratory analyses examined whether cognitive effects varied by exercise modality and cognitive–motor demand. Cognitive–motor demand was analysed as a continuous intervention-level index and grouped into low, moderate and high demand classes. To examine whether this index captured information distinct from physical dose, we assessed its correlations with physical-dose variables, cross-classified intervention arms by dose and demand level, and tested whether residual cognitive–motor demand, after adjustment for weekly volume, intervention duration, session duration and frequency, explained effect-size variability. An exploratory interaction between weekly volume and cognitive–motor demand was also tested.

## 3 Results

**Figure 1:**
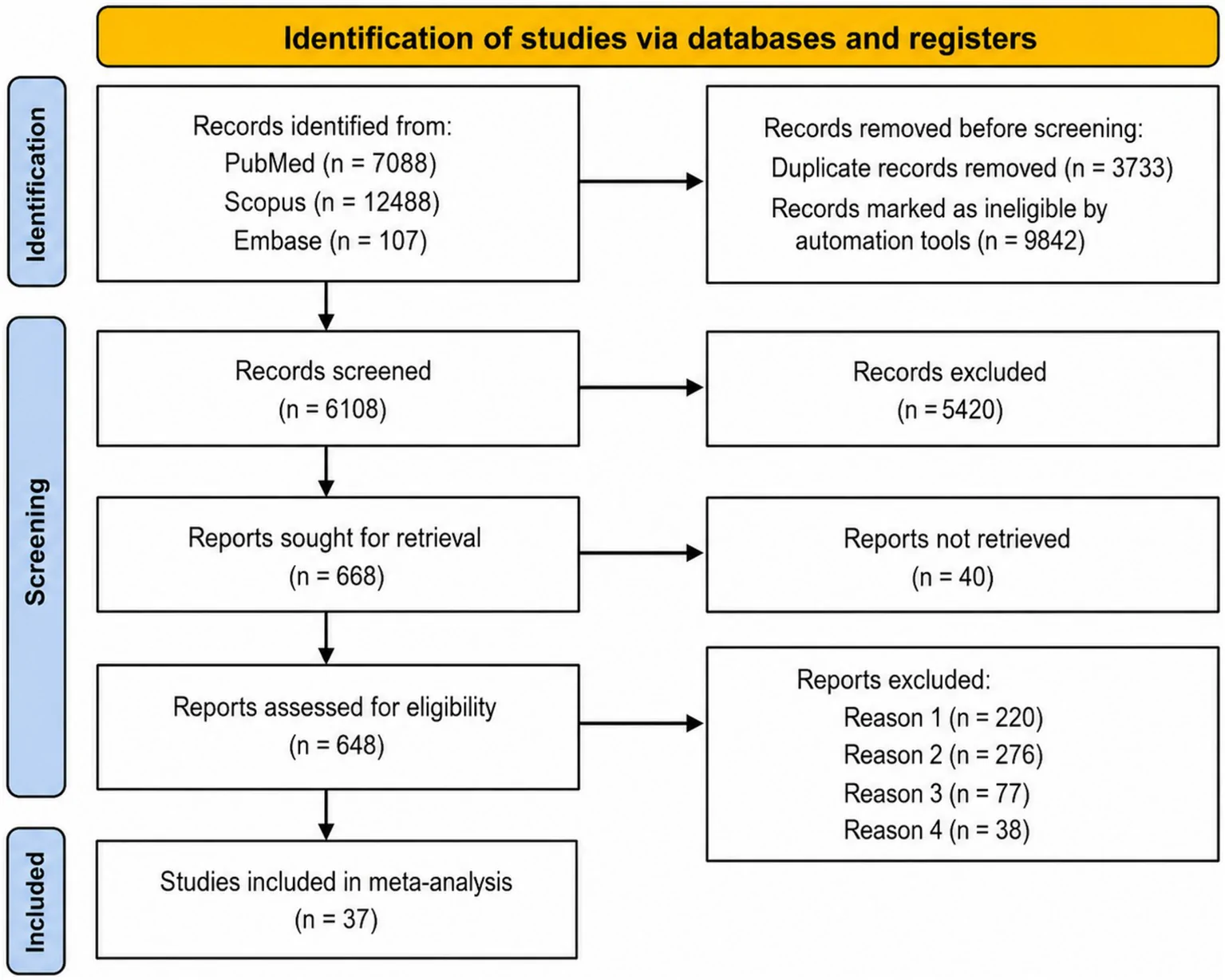
Prisma flow diagram of the meta-analysis. Reason 1 = study design (experiment, cross-over, no control group); Reason 2 = out of scope (wrong population, no physical activity); Reason 3 = no cognitive outcome; Reason 4 = unavailable data.

The study selection process is summarized in the PRISMA flow diagram (Figure 1) (Haddaway et al., 2022). The database search identified 19,683 records, of which 37 randomized controlled trials met the inclusion criteria, contributing 166 effect-size estimates across 11 cognitive outcomes.

**Figure 2:**
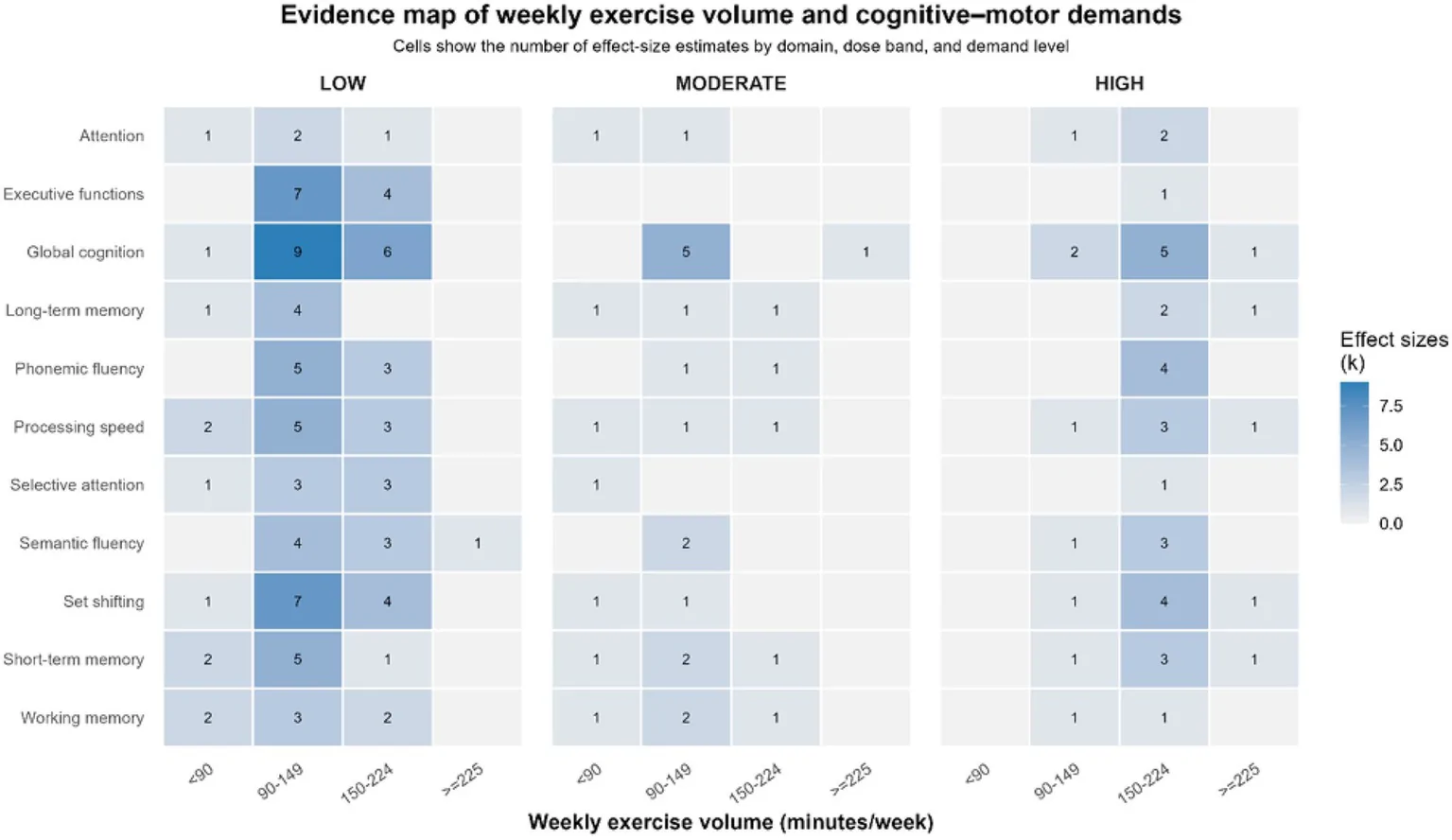
Evidence map of weekly exercise volume and cognitive–motor demands. Cells indicate the number of effect-size estimates contributing to each combination of cognitive domain, weekly exercise volume, and cognitive–motor demand level. Weekly exercise volume was calculated from intervention frequency and session duration, whereas cognitive–motor demand was classified using a structured intervention-level proxy score. The map shows that the evidence is unevenly distributed across the dose × demand space, with most data concentrated in low-demand and high-demand interventions and fewer observations in moderate-demand categories. This limited coverage suggests that current trials are not well positioned to isolate the independent or combined effects of physical dose and cognitive–motor demand.

Interventions were generally delivered two to three times per week, with sessions lasting 40–90 min and programmes most commonly lasting 12–24 weeks. Weekly exercise volume ranged from 60 to 300 min/week, with a median of 135 min/week. Exercise intensity was reported for approximately 62% of effect sizes and was not inferred when trial reports provided insufficient information. Interventions also varied in cognitive–motor demand, although their distribution across cognitive outcomes and demand levels was uneven. Full study and intervention characteristics are reported in Table 1, and the distribution of evidence across physical dose and cognitive–motor demand is shown in Figure 2.

**Table 1:** Characteristics included studies in the meta-analysis.

Study	Participants (Exp/Ctrl)	Age mean (years)	Female (%)	Intervention (*N*)	Intensity	Frequency (sessions/ weeks)	Session duration (min)	Total sessions
Antunes et al., 2015	22/23	67	NA	MICT (*n* = 22)	Moderate	3/24	60	72
Brown et al., 2021	67/32	69	54	HIIT (*n* = 33)	Vigorous	2/24	50	48
				MICT (*n* = 34)	Moderate			
Byun et al., 2024	40/41	68	75	AER (*n* = 40)	Light	3/12	40	36
Castillo Quezada et al., 2021	30/23	69	100	AER (*n* = 15)	Light	3/12	60	36
				ResT (*n* = 15)	Moderate	3/12	60	36
Chen et al., 2023	14/14	78	78	ResT (*n* = 14)	Mixed	2/12	50	24
Coelho-Júnior et al., 2020	22/15	67	100	Pow (*n* = 12)	Light	2/22	60	48
				ResT (*n* = 10)	Moderate	2/24	60	48
Coetsee and Terblanche, 2017	48/19	63	68	HIIT (*n* = 11)	Vigorous	3/16	60	48
				MICT (*n* = 13)	Vigorous	3/16	60	48
				ResT (*n* = 22)	Vigorous	3/16	60	48
Cunha et al., 2026	100/50	69	100	ResT (*n* = 50)	Moderate	3/12	60	36
				ST (*n* = 50)	Moderate	3/12	60	36
Dupuy et al., 2024	53/52	65	76	AER + COG (*n* = 53)	Moderate	5/24	30	120
Esmail et al., 2020	27/18	67	75	MICT (*n* = 15)	Moderate	3/12	60	36
				Dance (*n* = 12)	Moderate	3/12	60	36
Farinha et al., 2021	82/20	72	75	Combi (*n* = 29)	Mixed	2/28	45	56
				HIIT (*n* = 25)	Mixed	2/28	45	56
				MICT (*n* = 28)	Mixed	2/28	45	56
Frost et al., 2021	62/32	69	54	HIIT (*n* = 31)	Vigorous	2/24	50	48
				MICT (*n* = 31)	Moderate	2/24	50	48
Hola et al., 2024	36/27	70	88	Dance (*n* = 20)	N/A	2/12	90	24
				Tai Chi (*n* = 16)	N/A	2/12	90	24
Iuliano et al., 2015	20/20	67	60	ResT (*n* = 20)	Vigorous	3/12	30	36
Klusmann et al., 2010	80/76	73	100	AER (*n* = 80)	N/A	3/24	90	72
Kovacevic et al., 2020	41/23	72	60	HIIT (*n* = 21)	Vigorous	3/12	45	36
				MICT (*n* = 20)	Moderate	3/12	45	36
Kujawski et al., 2022	27/28	66	92	ResT (*n* = 27)	N/A	2/12	50	24
Li et al., 2024	23/25	67	88	Tai Chi (*n* = 23)	N/A	3/8	60	24
Lin et al., 2023	51/51	66	62	Tai Chi (*n* = 51)	N/A	3/24	60	72
Linde and Alfermann, 2014	31/13	67	60	AER (*n* = 15)	Mixed	3/16	60	48
				AER + COG (*n* = 16)			75	
Makino et al., 2021	310/105	72	47	AER (*n* = 104)	Light	2/26	60	52
		72	47	Combi (*n* = 104)	Mixed			
		72	47	ResT (*n* = 102)	Moderate			
Milot et al., 2026	9/11	65	N/A	AER (*n* = 9)	N/A	3/12	60	36
Muscari et al., 2010	53/56	70	48	MICT (*n* = 53)	Moderate	3/48	60	144
Park, 2022	28/28	67	50	AER (*n* = 28)	Mixed	3/5	40	15
Pellegrini-Laplagne et al., 2023	24/11	70	71	AER (*n* = 17)	Vigorous	3/12	30	36
				AER + COG (*n* = 7)	Light			
Alves et al., 2013	10/12	66	N/A	ST (*n* = 10)	Mixed	2/24	40	48
Shimada et al., 2018	53/53	70	48	Golf (*n* = 53)	Moderate	1/24	110	24
Su et al., 2021	35/35	65	52	Tai Chi (*n* = 35)	N/A	5/12	60	60
Tan et al., 2023	78/112	73	55,5	Combi (*n* = 37)	N/A	2/18	60	36
				Combi + COG (*n* = 41)				
Tarumi et al., 2022	36/37	69	75,5	AER (*n* = 36)	Mixed	3/24	30	72
Taylor-Piliae et al., 2010	64/28	69	70	Combi (*n* = 36)	Light	3/24	45	72
				Tai Chi (*n* = 28)				
Wan et al., 2022	26/24	65	58	Tai Chi (*n* = 26)	Mixed	3/24	60	72
Wang et al., 2024	60/60	67	79,2	Tai Chi (*n* = 60)	N/A	3/24	60	72
Wang et al., 2025	15/15	77	N/A	Tai Chi (*n* = 15)	Moderate	3/20	90	60
Welford et al., 2023	52/22	72	78,5	AER (*n* = 27)	Mixed	3/12	50	36
				Yoga (*n* = 25)				
Yang et al., 2025	45/45	67	67	Tai chi (*n* = 45)	Mixed	3/12	90	36
Ye et al., 2024	51/51	66	38	Martial art (*n* = 51)	N/A	3/24	60	72

In participants, EXP is for groups underwent exercise interventions, CTR is the comparator. Intervention’s abbreviation is MICT for moderate-intensity continuous training, HIIT for high-intensity interval training, AER is for aerobic, ResT is resistance training, Pow is power training, ST is strength training, combi is for combined training. N/A is not available information.

### 3.1 Overall effects across cognitive domains

Random-effects models showed the clearest evidence of benefit for global cognition. The pooled effect for global cognition was moderate and statistically significant (Hedges’ *g* = 0.55, 95% CI 0.38 to 0.73, *p* < 0.001; FDR-adjusted *p* < 0.001), although heterogeneity was substantial (*I*^2^ = 75.6%). This indicates an association between structured exercise and better global cognitive performance, but the magnitude and generalisability of this estimate should be interpreted in light of the observed heterogeneity.

For domain-specific outcomes, effect sizes were oriented so that positive values consistently indicated better cognitive performance in the exercise group relative to the comparator. Domain-specific effects were smaller and statistically uncertain. Executive functions showed a small positive estimate (*g* = 0.16, 95% CI approximately −0.00 to 0.33), as did phonemic fluency (*g* = 0.15, 95% CI − 0.02 to 0.31), set shifting (*g* = 0.14, 95% CI − 0.02 to 0.30), long-term memory (*g* = 0.11, 95% CI − 0.06 to 0.27), semantic fluency (*g* = 0.09, 95% CI − 0.08 to 0.26), working memory (*g* = 0.08, 95% CI − 0.04 to 0.20), processing speed (*g* = 0.06, 95% CI − 0.11 to 0.23), and short-term memory (*g* = 0.02, 95% CI − 0.16 to 0.20). Attention showed a small negative estimate (*g* = −0.08, 95% CI − 0.33 to 0.18), whereas selective attention was close to the null (*g* = −0.00, 95% CI − 0.27 to 0.26).

None of the domain-specific estimates survived false discovery rate correction. Overall, these findings indicate that the most robust signal was observed for global cognition, whereas effects on specific cognitive domains were small, heterogeneous and statistically uncertain. Importantly, after orienting all effect sizes in a common beneficial direction, the absence of robust domain-specific effects should be interpreted as imprecision and inconsistency rather than as evidence of adverse cognitive effects.

### 3.2 Risk of bias, publication bias and certainty of evidence

Risk of bias, publication bias and certainty of evidence were assessed separately from the main effect-size models. For each cognitive domain, risk-of-bias judgements are reported in Supplementary materials. Publication bias was explored using funnel plots and regression-based tests when the number of studies per outcome was sufficient. According to the GRADE assessment, the certainty of evidence was rated as very low for all cognitive outcomes. This was mainly due to the combined impact of risk of bias, between-study inconsistency, indirectness related to heterogeneous intervention and comparator types, and imprecision, particularly for domain-specific outcomes with small, pooled effects and confidence intervals crossing the null. Detailed assessments are provided in Supplementary Table S5.

### 3.3 Conventional physical-dose indicators

All outcomes with sufficient data were modelled using weekly exercise volume and intervention duration in weeks. Overall, conventional physical-dose indicators did not consistently explain between-study variability across cognitive outcomes. For global cognition, neither weekly exercise volume (*β* = 0.052, *p* = 0.705; FDR-adjusted *p* = 0.776) nor intervention duration (*β* = −0.060, *p* = 0.551; FDR-adjusted *p* = 0.758) was associated with effect-size variability.

Across domain-specific outcomes, no association between weekly exercise volume and cognitive effects survived false discovery rate correction. The closest uncorrected trends were observed for set shifting (*β* = 0.140, *p* = 0.094) and processing speed (*β* = 0.130, *p* = 0.104), but these were not statistically robust. Intervention duration also showed no robust association with cognitive outcomes; the closest uncorrected trends were observed for working memory (*β* = −0.119, *p* = 0.078), processing speed (*β* = −0.142, *p* = 0.096), and semantic fluency (*β* = −0.215, *p* = 0.117).

Spline-based models did not provide consistent evidence of non-linear dose–response associations, and no spline term survived false discovery rate correction. Overall, the available randomized trial evidence did not support a robust cognitive dose–response relationship based on weekly exercise volume or intervention duration alone. Because conventional physical-dose indicators did not consistently explain cognitive effects, we next examined whether intervention-level cognitive–motor demands captured complementary information about exercise content.

### 3.4 Cognitive–motor demands

Inter-rater agreement across the 56 independently coded intervention arms was high for motor complexity (quadratic-weighted *κ* = 0.811, 95% bootstrap CI 0.678–0.902), implicit cognitive demand (quadratic-weighted *κ* = 0.827, 95% CI 0.703–0.911), explicit cognitive training (*κ* = 0.879, 95% CI 0.486–1.000), the total cognitive–motor demand score (ICC[2,1], absolute agreement = 0.821, 95% CI 0.712–0.904), and the simplified demand class (quadratic-weighted *κ* = 0.882, 95% CI 0.792–0.946). Exact agreement was 69.6, 69.6, 98.2, 66.1, and 82.1%, respectively.

Exploratory analyses examined whether intervention-level cognitive–motor demands were associated with variability in cognitive effects and whether this dimension captured information distinct from conventional physical-dose indicators. The total cognitive–motor demand score showed only low-to-moderate correlations with weekly exercise volume, total intervention minutes, session duration, frequency, number of sessions and intervention duration. This suggests that cognitive–motor demand was not simply redundant with conventional physical-dose variables. Cross-classification of intervention arms also showed that interventions with similar physical dose could differ in cognitive–motor demand, supporting the distinction between physical exposure and qualitative task demands.

However, the overall cognitive–motor demand index was not significantly associated with effect sizes in the cross-domain model (*β* = 0.012, *p* = 0.419), nor was it associated with global cognition specifically (*β* = 0.016, *p* = 0.539). Outcome-specific associations for the overall index were not robust after false discovery rate correction. When cognitive–motor demand was tested alongside conventional physical-dose indicators, adding the total cognitive–motor demand score to weekly exercise volume and intervention duration did not improve model fit or explain additional effect-size variability. Similarly, the residual component of cognitive–motor demand not explained by physical-dose variables was not significantly associated with cognitive effects, and there was no evidence of an interaction between weekly volume and cognitive–motor demand.

Component-level analyses identified exploratory associations for set shifting. Motor complexity was negatively associated with oriented effect sizes (*β* = −0.523, *p* = 0.0004; FDR-adjusted *p* = 0.0047), whereas implicit cognitive stimulation was positively associated with oriented effect sizes (*β* = 0.542, *p* = 0.0002; FDR-adjusted *p* = 0.0016). These findings suggest that aggregating cognitive–motor demands into a single total score may obscure domain-specific patterns. However, because component scores were derived retrospectively from published descriptions and may overlap with exercise modality, supervision, novelty, task familiarity and reporting quality, these results should be considered hypothesis-generating rather than confirmatory. Detailed results are reported as Supplementary Table S6.

Categorical analyses comparing low, moderate and high cognitive–motor demand classes did not show robust omnibus effects after correction for multiple comparisons. The evidence map (Figure 2) showed sparse and uneven coverage across combinations of weekly volume and demand level, indicating that the available trials support exploratory mapping but not strong inference about the independent contribution of cognitive–motor demands. Overall, cognitive–motor demand appeared to describe a qualitative dimension of exercise content that was only partly overlapping with physical dose, but the current evidence did not show a general cognitive–motor demand effect across outcomes.

### 3.5 Sensitivity analysis excluding combined exercise–cognitive interventions

Studies combining both motor and cognitive exercise arms were less frequent and unevenly distributed across cognitive domains. In the analytic dataset, 13 of 166 effect-size estimates were removed when arms with explicit cognitive stimulation or cognitive training were excluded, leaving 154 motor-only effect sizes.

**Figure 3:**
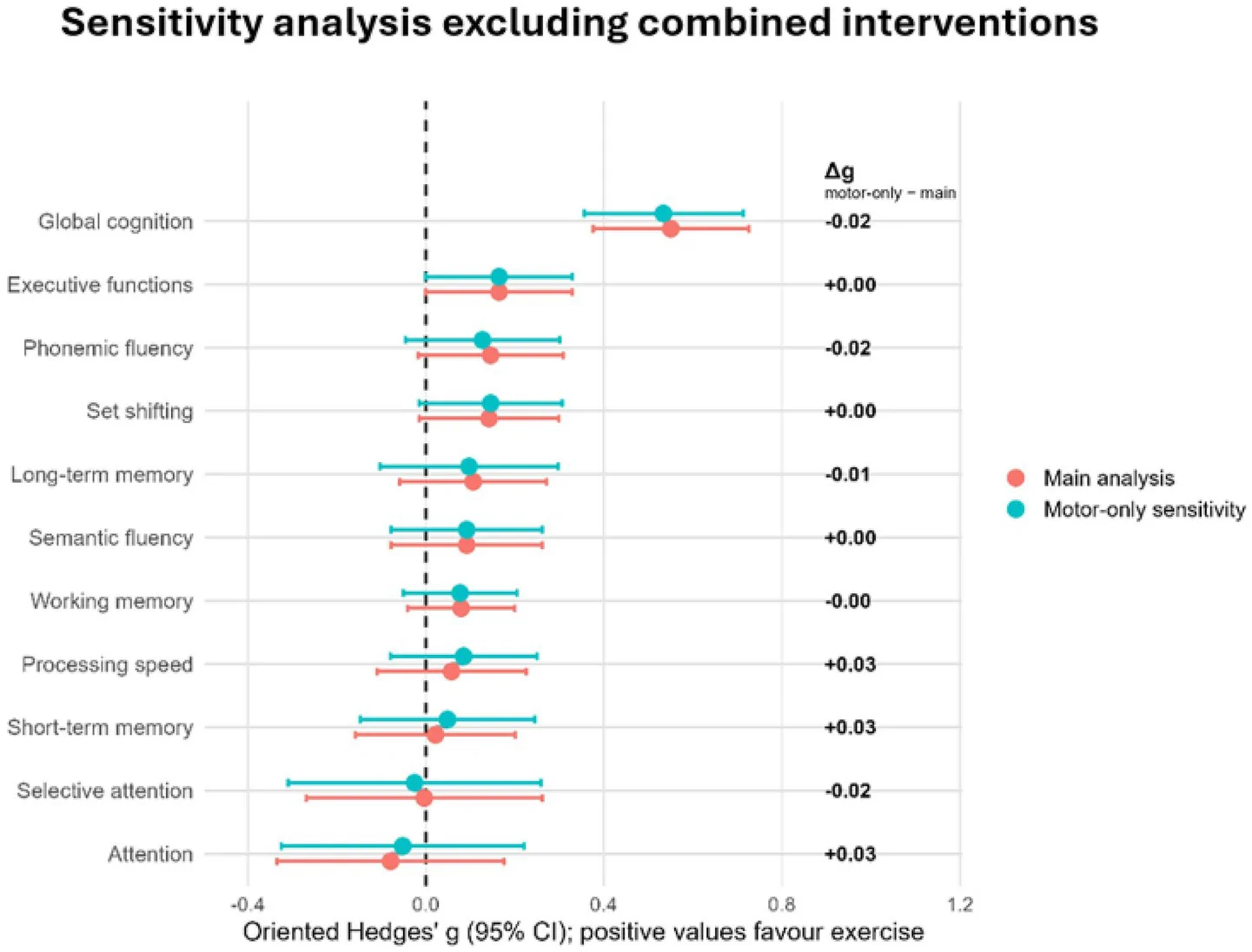
Sensitivity analysis excluding combined exercise–cognitive interventions. Pooled random-effects estimates from the main analysis are shown alongside estimates obtained after excluding exercise arms with an explicit cognitive-training or cognitive-stimulation component. Points represent pooled Hedges’ *g* values and horizontal bars indicate 95% confidence intervals; positive values favor exercise. The Δ*g* column reports the difference between the motor-only sensitivity estimate and the main estimate. Estimates were highly similar across domains, indicating that the observed pattern of results was robust to the exclusion of combined exercise–cognitive interventions.

Excluding these arms did not materially alter the pooled estimates as shown in Figure 3. For global cognition, the estimate changed only from *g* = 0.55 in the main analysis to *g* = 0.53 in the motor-only sensitivity analysis (Δ*g* = −0.02). Across domain-specific outcomes, absolute differences between the main and motor-only estimates ranged from 0.00 to 0.03. Executive functions remained unchanged, while small shifts were observed for processing speed, set shifting, attention, phonemic fluency, long-term memory, short-term memory and working memory.

These findings indicate that the main pooled estimates were not driven by the small number of combined exercise–cognitive interventions. However, combined motor-cognitive interventions remain conceptually distinct from motor-only exercise and should be analyzed separately when sufficient data are available.

## 4 Discussion

This systematic review with meta-analysis suggests that structured exercise is associated with better global cognitive outcomes in community-dwelling older adults but does not support a simple cognitive dose rule based on physical exposure alone. From a movement-science perspective, the main finding is that exercise interventions are rarely described in terms of the cognitive–motor operations they require. Current trials quantify how much exercise is delivered more consistently than how they specify how movement is organized, adapted, cued, varied or cognitively controlled. This limits the ability to understand exercise as a cognitive–motor intervention rather than as a generic physical exposure.

Consistent with previous meta-analyses, the clearest signal was observed for global cognition, whereas effects on specific cognitive domains were smaller and less stable (Gomes-Osman et al., 2018; Northey et al., 2018; Sanders et al., 2019; Falck et al., 2019). Most domain-specific estimates favored exercise, but their confidence intervals frequently crossed the null and none remained significant after correction for multiple testing. This pattern should not be interpreted as evidence that exercise benefits global cognition but has no effect on specific cognitive functions. Rather, it indicates that the evidence was more consistent for global measures and more uncertain for individual domains.

Several factors may account for this difference. Global cognitive measures combine performance across multiple cognitive processes and may therefore capture broad changes associated with exercise, including changes in physical function, cerebrovascular health, arousal, mood, self-efficacy and social engagement (Pagan et al., 2026). In contrast, improvements in a specific domain may require closer correspondence between the cognitive processes engaged during training and those assessed by the selected neuropsychological task. The domain-specific analyses also combined tests that differed in construct coverage, scoring and measurement sensitivity, and several domains were represented by relatively few studies. This combination of task heterogeneity and limited precision may have attenuated domain-specific pooled effects and reduced their robustness after false discovery rate correction. The larger estimate for global cognition should nevertheless be interpreted cautiously because heterogeneity was substantial and the certainty of evidence was very low. The absence of a consistent physical dose–response relationship should not be interpreted as evidence that exercise dose is irrelevant. The available interventions covered a relatively restricted and clustered range of prescriptions: most programs involved two or three weekly sessions, sessions of broadly comparable duration and interventions lasting between 12 and 24 weeks. Consequently, the meta-regression models had limited information at the extremes of the dose distribution from which to identify either linear gradients or non-linear thresholds. Moreover, dose was compared across trials rather than experimentally manipulated within studies. Differences in weekly volume and intervention duration were therefore accompanied by differences in modality, supervision, participant characteristics and control conditions. The analyses were also based primarily on prescribed dose, whereas adherence, progression and the exercise actually completed were reported less consistently. These limitations may have obscured a dose–response relationship even if one exists (Burnet et al., 2019). The present findings therefore indicate that current trials do not support a precise cognitive prescription based on weekly volume or intervention duration alone, rather than demonstrating that the amount of exercise has no relevance for cognitive outcomes.

This distinction is particularly relevant for movement science. Exercise can be considered not only as a physiological exposure, but also as a structured motor behavior requiring perception, action selection, coordination, monitoring and adaptation. From this perspective, the cognitive relevance of an exercise intervention may depend partly on the operations required to perform and adapt movement. These findings are therefore relevant to the study of adaptation in ageing because many exercise interventions require older adults to continuously plan, monitor and adjust movement under changing task constraints, yet these features are rarely isolated from conventional physical-dose parameters. Current trials often compare broad exercise programs that differ simultaneously in modality, intensity, duration, supervision, progression and task content. As a result, it remains difficult to determine whether cognitive outcomes are driven by the amount of exercise performed, by the cognitive–motor characteristics of the task, or by their interaction.

The distinction between physical exposure and task demands is consistent with existing theoretical frameworks. Voelcker-Rehage and Niemann (2013) distinguished metabolic from coordinative exercise demands, suggesting that different physical activities may engage partly distinct cognitive and neural mechanisms. Herold et al. (2018) further clarified that motor-cognitive interventions differ depending on whether cognitive and motor components are performed separately, simultaneously or in an integrated way. These frameworks support the rationale of the present review: exercise dose for cognitive ageing cannot be fully captured by FITT variables alone if the cognitive–motor structure of the activity is ignored.

The cognitive–motor demand index as an intervention-level measure was used as a structured surrogate measure of a latent variable that is rarely explicitly measured in exercise trials. The poor-to-moderate correlation between the index and weekly volume, session duration, and intervention duration indicated that this index tapped into a feature of exercise that could not be solely captured by the physical stimulus. While this distinction is made merely on a descriptive basis, this does not necessarily mean that the demand index has incremental validity. The index score was calculated based on a post-hoc analysis of the description of interventions in literature and hence its reliability depended on how comprehensively and precisely such interventions were described in the trials.

The index was further related to intervention features like modality, supervision, novelty, and task familiarity, which were not experimentally manipulated separately in the trials included in the analysis. Moreover, combining different cognitive-motor features into one variable may mask domain-specific or opposite correlations. This explains the lack of improvement of the predictive power of models when using the total index in comparison with conventional physical-dose measures. However, the non-incremental value of the index cannot be regarded as the proof that cognitive-motor task demands have no impact. It is simply because the trials conducted currently do not provide enough information on them.

The exploratory component-level findings for set shifting raise the possibility that cognitive–motor demands are multidimensional rather than reducible to a single overall score. Set shifting depends on flexible updating, task switching and control processes (Egner, 2023), and may therefore be sensitive to task features such as adaptability, response selection, cueing or changing task constraints. However, the opposing associations observed for motor complexity and implicit cognitive stimulation caution against a simple interpretation whereby greater cognitive–motor demand is necessarily better. Because these components were also closely associated with exercise modality, their opposing coefficients may reflect modality-related confounding rather than separable effects of motor complexity and implicit cognitive stimulation. These findings cannot determine which specific task features are beneficial, neutral or confounded with other intervention characteristics. They should therefore be interpreted as hypothesis-generating, particularly because the component scores were likely correlated with modality, supervision, novelty, reporting completeness and task familiarity.

The sensitivity analysis excluding combined exercise–cognitive interventions also help distinguish explicit cognitive stimulation delivered as an add-on from cognitive–motor demands embedded within the exercise task itself. Excluding arms that added cognitive training, computerized tasks or structured mental activity did not materially alter pooled estimates, indicating that the main findings were not driven by these combined interventions. However, this should not be interpreted as evidence that cognitive engagement is irrelevant. Motor-only intervention can still be cognitively demanding when the movement task requires balance control, sequencing, coordination, adaptation to external cues, skill learning or continuous sensorimotor monitoring. Conversely, an added cognitive component does not necessarily imply that cognitive demands are integrated into the movement task. This distinction is important because sequential exercise-plus-cognitive training and integrated dual-task training may rely on partly different mechanisms and may have different implications for transfer (Tait et al., 2017).

This broader view is supported by related empirical literature. Research on open versus closed-skill exercise suggests that adaptability, environmental variability and decision-making may be cognitively relevant features of physical activity (Gu et al., 2019). Motor-cognitive and dual-task training frameworks further show that cognitive and motor demands can be combined in different formats, including sequential, simultaneous and integrated approaches, with potentially different effects on cognitive transfer (Tait et al., 2017; Herold et al., 2018). Evidence on experience-dependent plasticity also suggests that qualitative features of movement experience, including novelty, learning demands and multisensory stimulation, may be relevant when considering exercise effects on cognition (Kramer et al., 2004). This view is consistent with the proposal that exercise, sport and performance arts may influence cognition through shared processes such as skill acquisition, action selection, monitoring and adaptation (Tomporowski and Pesce, 2019).

Accordingly, interventions such as dance, Tai Chi, yoga, martial arts, golf and some multicomponent programs should not be treated merely as alternative exercise types. They may represent qualitatively different cognitive–motor experiences that are not captured by minutes per week or physiological intensity alone. This has implications for trial design. Randomized controlled trials remain essential for estimating whether exercise interventions produce cognitive benefits. However, most trials in this field were not designed to disentangle physical exposure from cognitive–motor demands. They typically compare broad exercise programs that differ simultaneously in duration, intensity, modality, supervision, progression, social context and task content. More informative designs would match interventions for weekly volume and physiological intensity while systematically varying specific cognitive–motor features, such as task variability, sequencing, cueing, balance demands, decision-making or adaptive progression. Pragmatic trials could also compare interventions matched for duration and intensity but differing in the cognitive and motor operations required by the task.

From a translational perspective, these findings caution against a simplistic prescription model in which cognitive benefit is assumed to increase within minutes per week alone. Duration, frequency and intensity remain essential for safety, feasibility and physiological adaptation. However, when cognition is an intended outcome, intervention design should consider not only how much exercise is delivered, but also which cognitive–motor task features are embedded within the activity. These demands should be progressed adaptively, not maximized indiscriminately, especially in older adults with frailty, low confidence or balance limitations. In this sense, the aim is not to prescribe “more complexity” as a universal rule, but to identify which movement structures, task demands and adaptive control processes are appropriate for specific cognitive outcomes and participant profiles.

Several limitations should be acknowledged. First, the cognitive–motor demand index was a structured intervention-level classification, not a validated scale or a direct measure of cognitive load. Because it was derived from published descriptions, it may partly reflect reporting quality rather than the actual demands experienced by participants. Demand scores may also be confounded with modality, supervision, social interaction, novelty, instructor expertise and task familiarity. The present findings therefore support the need for better reporting and experimental manipulation of cognitive–motor task features rather than confirming a general cognitive–motor dose effect.

Second, the interpretation of these analyses is limited by the absence of an established metric for cognitive dose. Unlike physical exercise, where dose can be described using frequency, duration, volume and intensity, cognitive exposure is more difficult to quantify. This challenge is not specific to exercise trials. Meta-analyses of cognitive stimulation or cognitive training have also highlighted that interventions vary in task type, difficulty, adaptivity, feedback, progression and transfer demands, without reducing these features to a shared dose scale (Lampit et al., 2014; Basak et al., 2020). Therefore, the lack of a clear cognitive–motor demand effect in the present review should not be interpreted as evidence that cognitive demands are irrelevant. Rather, it indicates that current trials are not yet designed or reported in a way that allows cognitive exposure to be isolated from physical dose.

Third, classification of neuropsychological tests into cognitive domains necessarily involved some judgement, although domain assignments were guided by established neuropsychological and neuroscientific references. Some tests involve multiple processes and assigning them to a single domain may obscure cross-domain cognitive demands. This issue is particularly relevant for executive function, processing speed and set-shifting outcomes. Some cognitive domains included relatively few effect-size estimates, limiting the stability of moderator models and increasing the risk of overfitting. Interventions also differed across multiple correlated features, including modality, intensity, supervision, social context, novelty, progression and participant characteristics. Finally, although effect sizes were oriented so that positive values consistently favored the exercise group, this required judgement for outcomes based on completion times, errors or accuracy. Transparent reporting of outcome direction remains important in exercise–cognition meta-analyses. These limitations are not only methodological weaknesses; they also identify the changes needed in the next generation of trials.

Overall, the present review suggests that the field should move beyond asking only whether exercise benefits cognition or how many minutes per week are required. For cognitive ageing, a more informative question is how physical exposure, movement structure, task demands and adaptive control processes jointly shape cognitive outcomes. Future trials should therefore specify, manipulate and report both physical dose and cognitive–motor task features. This would help move the field from broad modality comparisons toward a more mechanistic understanding of how movement-based interventions engage cognitive processes in ageing.

## 5 Conclusion

Structured exercise was associated with better global cognitive performance in community-dwelling older adults, whereas domain-specific effects were generally favorable in direction but small, heterogeneous and statistically uncertain. The findings do not support a simple cognitive dose rule based on minutes per week, frequency or intervention duration alone. At the same time, they do not establish cognitive–motor demand as a confirmed independent dose dimension. Rather, they indicate that current randomized trials are not yet designed or reported in a way that allows physical exposure to be clearly separated from the cognitive and motor demands embedded in exercise tasks.

From a movement-science perspective, these findings suggest that exercise interventions for cognitive ageing should be described not only by how much exercise is delivered, but also by how movement is structured, varied, cued, monitored and adapted. Future trials should therefore specify, manipulate and report both conventional physical-dose parameters and cognitive–motor task features, including coordination, sequencing, balance control, attentional engagement, decision-making and adaptive progression. This would help move the field from the broad question of whether exercise benefits cognition to a more mechanistic and clinically useful question: which movement-based exercise features matter, for which cognitive outcomes, and for whom.

## Data Availability

The original contributions presented in the study are included in the article/Supplementary material, further inquiries can be directed to the corresponding author.
