# Supplementary material for: Rethinking exercise dose for cognitive ageing: a systematic review and meta-analysis of physical dose and cognitive–motor demands
Source: Front Psychol. 2026 Aug 13;17:1906526. doi: 10.3389/fpsyg.2026.1906526 (PMC13518203; doi:10.3389/fpsyg.2026.1906526)
Supplement: Supplementary file 1 [file Supplementary_file_1.docx]

Contents

[Table 1. Strings Search used in this review	5](#_Toc230606250)

[Table 2. Cognitive data categorization	7](#_Toc230606251)

[Standardization of Exercise Intensity and categorization of Sport and Physical activities.	9](#_Toc230606252)

[Table 3. 1 Exercise intensity classification	9](#_Toc230606253)

[Table 3.2 Sport modality classification	9](#_Toc230606254)

[Coding of cognitive–motor complexity	11](#_Toc230606255)

[Table 4. Operationalization of intervention cognitive–motor complexity	11](#_Toc230606256)

[Table 5 GRADE Summary of Findings	13](#_Toc230606257)

[Table 6 Relationship between conventional physical dose and cognitive–motor demand	16](#_Toc230606258)

[6.1. Spearman correlations	16](#_Toc230606259)

[6.2 Cross-classification of effect sizes by physical dose and cognitive–motor demand	16](#_Toc230606260)

[6.3 Model fit: physical dose vs cognitive–motor demand	17](#_Toc230606261)

[6.4 Incremental value of cognitive–motor demand beyond physical dose	17](#_Toc230606262)

[6.5 Exploratory set-shifting models	18](#_Toc230606263)

[Outcome 1: Attention	20](#_Toc230606264)

[1.1 Results from meta-analysis	20](#_Toc230606265)

[1.2 Forest Plot	20](#_Toc230606266)

[1. 3 Funnel Plot	20](#_Toc230606267)

[1.4 Asymmetry test	21](#_Toc230606268)

[1.5 Risk of Bias	21](#_Toc230606269)

[1.6 Leave one out plot	21](#_Toc230606270)

[1.7 Forest Plot with sub-group sport categories	22](#_Toc230606271)

[1.8 Sport effect estimates table	22](#_Toc230606272)

[Outcome 2: Executive Functions	23](#_Toc230606273)

[2.1 Results from meta-analysis	23](#_Toc230606274)

[2.2 Forest Plot	23](#_Toc230606275)

[2.3 Funnel Plot	24](#_Toc230606276)

[2.4 Asymmetry test	24](#_Toc230606277)

[2.5 Risk of Bias	24](#_Toc230606278)

[2.6 Leave one out plot	25](#_Toc230606279)

[2.7 Forest Plot with sub-group sport categories	26](#_Toc230606280)

[2.8 Sport effect estimates table	26](#_Toc230606281)

[Outcome 3: Global Cognitive Functioning	27](#_Toc230606282)

[3.1 Results from meta-analysis	27](#_Toc230606283)

[3.2 Forest Plot	27](#_Toc230606284)

[3.3 Funnel plot	28](#_Toc230606285)

[3.4 Asymmetry test	28](#_Toc230606286)

[3.5 Risk of Bias	28](#_Toc230606287)

[3.6 Leave one out plot	29](#_Toc230606288)

[3.7 Forest Plot with sub-group sport categories	30](#_Toc230606289)

[3.8 Sport effect estimates table	31](#_Toc230606290)

[Outcome 4: Long Term Memory	32](#_Toc230606291)

[4.1 Results from meta-analysis	32](#_Toc230606292)

[4.2 Forest Plot	32](#_Toc230606293)

[4.3 Funnel Plot	32](#_Toc230606294)

[4.4 Asymmetry test	33](#_Toc230606295)

[4.5 Risk of Bias	33](#_Toc230606296)

[4.6 Leave one out plot	33](#_Toc230606297)

[4.7 Forest Plot with sub-group sport categories	34](#_Toc230606298)

[4.8 Sport effect estimates table	34](#_Toc230606299)

[Outcome 5: Phonemic Fluency	35](#_Toc230606300)

[5.1 Results from meta-analysis	35](#_Toc230606301)

[5.2 Forest Plot	35](#_Toc230606302)

[5.3 Funnel Plot	36](#_Toc230606303)

[5.4 Asymmetry test	36](#_Toc230606304)

[5.5 Risk of Bias	36](#_Toc230606305)

[5.6 Leave one out plot	37](#_Toc230606306)

[5.7 Forest Plot with sub-group sport categories	38](#_Toc230606307)

[5.8 Sport effect estimates table	39](#_Toc230606308)

[Outcome 6: Processing Speed	40](#_Toc230606309)

[6.1 Results from meta-analysis	40](#_Toc230606310)

[6.2 Forest Plot	40](#_Toc230606311)

[6.3 Funnel Plot	40](#_Toc230606312)

[6.4 Asymmetry test	41](#_Toc230606313)

[6.5 Risk of Bias	41](#_Toc230606314)

[6.6 Leave one out plot	41](#_Toc230606315)

[6.7 Forest Plot with sub-group sport categories	42](#_Toc230606316)

[6.8 Sport effect estimates table	43](#_Toc230606317)

[Outcome 7: Selective Attention	44](#_Toc230606318)

[7.1 Results from meta-analysis	44](#_Toc230606319)

[7.2 Forest Plot	44](#_Toc230606320)

[7.3 Funnel Plot	44](#_Toc230606321)

[7.4 Asymmetry test	45](#_Toc230606322)

[7.5 Risk of Bias	45](#_Toc230606323)

[7.6 Leave one out plot	45](#_Toc230606324)

[7.7 Forest Plot with sub-group sport categories	46](#_Toc230606325)

[7.8 Sport effect estimates table	46](#_Toc230606326)

[Outcome 8: Semantic Fluency (secondary analysis)	47](#_Toc230606327)

[8.1 Results from meta-analysis	47](#_Toc230606328)

[8.2 Forest Plot	47](#_Toc230606329)

[8.3 Funnel Plot	47](#_Toc230606330)

[8.4 Asymmetry test	48](#_Toc230606331)

[8.5 Risk of Bias	48](#_Toc230606332)

[8.6 Leave one out plot	48](#_Toc230606333)

[8.7 Forest Plot with sub-group sport categories	49](#_Toc230606334)

[8.8 Sport effect estimates table	50](#_Toc230606335)

[Outcome 9: Set Shifting	51](#_Toc230606336)

[9.1 Results from meta-analysis	51](#_Toc230606337)

[9.2 Forest Plot	51](#_Toc230606338)

[9.3 Funnel Plot	52](#_Toc230606339)

[9.4 Asymmetry test	52](#_Toc230606340)

[9.5 Risk of Bias	52](#_Toc230606341)

[9.6 Leave one out plot	53](#_Toc230606342)

[9.7 Forest Plot with sub-group sport categories	54](#_Toc230606343)

[9.8 Sport effect estimates table	55](#_Toc230606344)

[Outcome 10: Short-Term Memory (secondary analysis)	56](#_Toc230606345)

[10.1 Results from meta-analysis	56](#_Toc230606346)

[10.2 Forest Plot	56](#_Toc230606347)

[10.3 Funnel Plot	56](#_Toc230606348)

[10.4 Asymmetry test	57](#_Toc230606349)

[10.5 Risk of Bias	57](#_Toc230606350)

[10.6 Leave one out plot	57](#_Toc230606351)

[10.7 Forest Plot with sub-group sport categories	58](#_Toc230606352)

[10.8 Sport effect estimates table	59](#_Toc230606353)

[Outcome 11: Working Memory	60](#_Toc230606354)

[11.1 Results from meta-analysis	60](#_Toc230606355)

[11.2 Forest Plot	60](#_Toc230606356)

[11.3 Funnel Plot	60](#_Toc230606357)

[11.4 Asymmetry test	61](#_Toc230606358)

[11.5 Risk of Bias	61](#_Toc230606359)

[11.6 Leave one out plot	61](#_Toc230606360)

[11.7 Forest Plot with sub-group sport categories	62](#_Toc230606361)

[11.8 Sport effect estimates table	62](#_Toc230606362)

# Table 1. Strings Search used in this review

The table below presents the search strategies used in the electronic databases. Both the final advanced search queries and selected sensitive search strings explored during the development of the strategy are reported to ensure transparency and facilitate reproducibility of the search process.

| Database | Advanced | Sensitive |
| --- | --- | --- |
| PubMed – Medline | (  ("Aged"[Mesh] OR "Aged, 80 and over"[Mesh]  OR elderly[tiab] OR "older adult*"[tiab] OR "older people"[tiab])  )  AND  (  ("Exercise"[Mesh] OR "Motor Activity"[Mesh] OR "Physical Exertion"[Mesh]  OR exercise[tiab] OR "physical activity"[tiab]  OR "aerobic training"[tiab] OR "resistance training"[tiab]  OR "strength training"[tiab] OR "multicomponent training"[tiab])  )  AND  (  ("Cognition"[Mesh]  OR "Executive Function"[Mesh]  OR "Memory"[Mesh]  OR cognition[tiab]  OR cognitive[tiab]  OR "executive function*"[tiab]  OR memory[tiab]  OR attention[tiab]  OR "processing speed"[tiab])  )  AND  (  (randomized controlled trial[pt]  OR controlled clinical trial[pt]  OR randomized[tiab]  OR randomised[tiab]  OR randomly[tiab]  OR trial[ti])  )  AND  (  english[lang]  )  NOT  (  animals[mh] NOT humans[mh]  )  NOT  (  preprint[pt]  ) | "Aged" [Mesh] AND "Exercise"[Mesh] AND "Cognition "[Mesh] |
| Scopus – Web of Science | TITLE-ABS-KEY(  elderly OR "older adult*" OR "older people" OR aged  )  AND  TITLE-ABS-KEY(  exercise OR "physical activity" OR "aerobic training"  OR "resistance training" OR "strength training"  OR "multicomponent training"  )  AND  TITLE-ABS-KEY(  cognition OR cognitive OR "executive function*"  OR memory OR attention OR "processing speed"  )  AND  TITLE-ABS-KEY(  randomized OR randomised OR randomly  OR "randomized controlled trial"  OR "controlled trial"  ) | physical activity AND elderly OR older adults AND cognitive |
| Embase | ('aged'/exp OR elderly:ti,ab)  AND  ('exercise'/exp OR 'physical activity'/exp)  AND  ('cognition'/exp OR cognitive:ti,ab)  AND  ('randomized controlled trial'/exp)  AND [human]/lim  AND [english]/lim  NOT 'preprint'/it | physical activity OR exercise AND elderly OR older adults AND cognitive OR cognition AND randomized controlled trial |

# Table 2. Cognitive data categorization

| Domain | Definition | Neuropsychological test included | outcome |
| --- | --- | --- | --- |
| Executive Functions | performance in interference control tasks requiring inhibition of a prepotent response | Stroop inhibition condition Flanker task Simon task | number of inhibition errors |
| Global Cognition | composite scores obtained from standardized cognitive screening tools | Montreal Cognitive Assessment (MoCA) Mini-Mental State Examination (MMSE) | total score (points) |
| Long Term Memory | verbal episodic memory including learning and delayed recall | Rey Auditory Verbal Learning Test – total learning score (RAVLT total) California Verbal Learning Test – Long Delay Free Recall (CVLT-LDFR) | number of correctly recalled words (score) |
| Phonemic Verbal Fluency | number of correct words produced within a fixed time interval | FAS test Letter Fluency Test Letter P task | number of words (score) |
| Processing Speed | performance in tasks requiring rapid visual scanning, symbol matching, or colour naming. | Trail Making Test A (TMT-A) Stroop Color / Color naming condition Digit Symbol Substitution Test | completion time (seconds) |
| Selective Attention | accuracy or error rate in tasks requiring stimulus selection or response monitoring. | Stroop task (errors) | Percentage of correct response |
| Semantic Verbal Fluency | number of category-related words generated within a fixed time interval | Category Fluency Test Animal naming task | number of correct words (score) |
| Set Shifting | time or accuracy in tasks requiring alternation between cognitive sets | Trail Making Test B (TMT-B) | completion time (seconds) |
| Short-Term Memory | recall or span in immediate memory tasks. | Digit Span Forward | span length |
| Working Memory | performance in tasks requiring both storage and mental manipulation of information | Digit Span Backward N-Back tasks (1-back, 2-back) | span score |
| Processing Speed | performance in tasks requiring rapid visual scanning, symbol matching, or colour naming. | Trail Making Test A (TMT-A)  Stroop Color / Color naming condition  Digit Symbol Substitution Test  WAIS Digit Symbol | completion time (seconds) |

# Standardization of Exercise Intensity and categorization of Sport and Physical activities.

## Table 3. 1 Exercise intensity classification

Because the included studies reported exercise intensity using heterogeneous metrics (e.g., percentage of maximal heart rate, VO₂max, Borg rating of perceived exertion, or qualitative descriptions), exercise intensity was classified into standardized categories.

When intensity was explicitly reported, the original metric provided by the study was used. When necessary, reported values were converted to comparable categories based on established exercise physiology thresholds.

Exercise intensity was categorized into three levels. When studies only reported qualitative descriptions (e.g., light, moderate, high intensity training), classification followed the authors’ definitions when consistent with these thresholds.

If multiple intensity levels were implemented within the same program (e.g., interval protocols), we adopted the classification “mixed”.

| **Intensity level** | **%HRmax** | **%VO₂max** | **METs** | **RPE Borg (6–20)** |
| --- | --- | --- | --- | --- |
| **Low** | <64% | <46% | <3 | <11 |
| **Moderate** | 64–76% | 46–63% | 3–6 | 12–13 |
| **Vigorous** | 77–95% | 64–90% | 6–9 | 14–17 |

## Table 3.2 Sport modality classification

To reduce between-study heterogeneity and allow meaningful subgroup analyses, individual sport interventions were standardized into physiologically coherent sport modality categories.

Each intervention was classified according to the primary physiological and motor characteristics of the activity, rather than the specific sport label reported in the original study.

When interventions combined multiple components (e.g., aerobic + resistance training), classification was based on the dominant training stimulus described in the protocol.

If a study included multiple experimental arms featuring different sport modalities, each arm was treated as a separate intervention comparison within the meta-analysis.

| **Category** | **Sport type reported in studies** | **Description** |
| --- | --- | --- |
| **Aerobic Continous** | Moderate-Intensity Continuous Training (MICT), Aerobic | Continuous aerobic exercise performed at moderate intensity aimed at improving cardiorespiratory fitness (e.g., treadmill exercise, cycling). |
| **Interval High Intensity** | High Intensity Interval Training (HIIT) | Interval-based aerobic training characterized by repeated bouts of high-intensity effort interspersed with recovery periods. |
| **Resistance training** | Resistance Training | Resistance-based exercise programs using external loads (machines, free weights, bands) to improve muscular strength. |
| **Strength** | Strength Training | Strength-oriented training interventions focused on muscle force production. |
| **Multicomponent Combined** | Combined training, Power | Programs combining multiple training components (e.g., aerobic, resistance, balance, or power training). |
| **Mind Body Skill** | Dance, Tai Chi, Golf, Martial Art | Activities requiring coordination, balance, motor learning, and cognitive engagement. |
| **Mind Body Flexibility** | Tai Chi (Baduanjin), Yoga | Low-intensity mind–body activities emphasizing flexibility, breathing control, and postural stability. |

# Coding of cognitive–motor complexity

To characterize the cognitive demands embedded in each intervention, we coded each intervention arm according to three components: motor complexity, implicit cognitive enrichment, and explicit cognitive training. Motor complexity captured the coordinative, balance, sequencing, and motor-learning demands of the physical task. Implicit cognitive enrichment captured cognitive demands embedded within the exercise itself, such as attention, cue response, rhythm, spatial orientation, rule following, or adaptation to changing task constraints. Explicit cognitive training captured the presence of structured cognitive exercises delivered together with, before, or after the physical intervention.

Each component was scored using the information reported in the original intervention descriptions. The component scores were then summed to derive an overall cognitive–motor complexity score, with higher values indicating interventions with richer cognitive–motor demands. Interventions were also grouped into broader complexity classes to support descriptive and exploratory moderator analyses. Coding was conservative: when intervention details were insufficient, no additional complexity features were inferred.

# Table 4. Operationalization of intervention cognitive–motor complexity

| **Intervention profile** | **Motor complexity** | **Implicit cognitive enrichment** | **Explicit cognitive training** | **Revised total score** | **Revised class** | **Simplified class** |
| --- | --- | --- | --- | --- | --- | --- |
| Continuous aerobic exercise / MICT / simple walking or cycling | 0 | 0 | 0 | 0 | Low physical-only | Low |
| HIIT or aerobic exercise with intensity variation but limited coordinative demand | 1 | 0 | 0 | 1 | Low physical-only | Low |
| Resistance training / strength training | 1 | 1 | 0 | 2 | Low–moderate | Low |
| Multicomponent physical training without explicit cognitive training | 2 | 2 | 0 | 4 | Moderate | Moderate |
| Aerobic exercise + explicit cognitive training | 0 | 0 | 4 | 4 | Moderate / cognitive-added | Moderate |
| Mind–body flexibility exercise, e.g., yoga or basic tai chi | 3 | 3 | 0 | 6 | High | High |
| Skill-based mind–body or sport-like interventions, e.g., dance, martial arts, golf, skill-based tai chi | 4 | 4 | 0 | 8 | Very high | High |
| Multicomponent physical training + explicit cognitive training | 2 | 2 | 4 | 8 | Very high | High |

# Table 5 GRADE Summary of Findings

**Author(s):** Systematic review with meta-analysis author

**Question:** Physical exercise or sport-based interventions compared to Usual care or no intervention for healthy older adults

**Setting:** Community-dwelling older adults

**Bibliography:**

| **Certainty assessment** | | | | | | | **№ of patients** | | **Effect** | | **Certainty** | **Importance** |
| --- | --- | --- | --- | --- | --- | --- | --- | --- | --- | --- | --- | --- |
| **№ of studies** | **Study design** | **Risk of bias** | **Inconsistency** | **Indirectness** | **Imprecision** | **Other considerations** | **Physical exercise or sport-based interventions** | **Usual care or no intervention** | **Relative (95% CI)** | **Absolute (95% CI)** |  |  |
| **Attention (assessed with: Digit Symbol Test)** | | | | | | | | | | | | |
| 5 | randomised trials | serious^a^ | serious^a,b^ | not serious | serious^c^ | none^d^ | 445 | 459 | - | SMD **0.08 SD lower** (0.33 lower to 0.18 higher) | ⨁◯◯◯ Very low^a,b,c,d^ | IMPORTANT |
| **executive function (assessed with: Flanker score, Simon task)** | | | | | | | | | | | | |
| 6 | randomised trials | very serious^e^ | not serious | not serious | very serious^f^ | none | 280 | 296 | - | SMD **0.16 SD lower** (0 to 0.32 higher) | ⨁◯◯◯ Very low^e,f^ | CRITICAL |
| **Global Functioning (assessed with: MoCA, MMSE)** | | | | | | | | | | | | |
| 19 | randomised trials | serious^g^ | serious^b^ | not serious | not serious | publication bias strongly suspected^d^ | 1121 | 1254 | - | SMD **0.55 SD higher** (0.38 higher to 0.73 higher) | ⨁◯◯◯ Very low^b,d,g^ | CRITICAL |
| **Long term memory (assessed with: CVLT, RAVLT)** | | | | | | | | | | | | |
| 6 | randomised trials | serious^g^ | not serious^b^ | serious^h^ | serious^c^ | none | 517 | 534 | - | SMD **0.11 SD higher** (0.06 lower to 0.27 higher) | ⨁◯◯◯ Very low^b,c,g,h^ | IMPORTANT |
| **Phonological Fluency (assessed with: Letter Fluency Test)** | | | | | | | | | | | | |
| 8 | randomised trials | serious^g^ | serious^b^ | serious^h^ | serious^c^ | none | 714 | 718 | - | SMD **0.15 SD higher** (0.02 lower to 0.3 higher) | ⨁◯◯◯ Very low^b,c,g,h^ | IMPORTANT |
| **Processing speed (assessed with: TMT A, (s) Stroop Test Color (s))** | | | | | | | | | | | | |
| 11 | randomised trials | serious^g^ | serious^b^ | serious^h^ | serious^c^ | none | 735 | 744 | - | SMD **0.09 SD lower** (0.26 lower to 0.09 higher) | ⨁◯◯◯ Very low^b,c,g,h^ | CRITICAL |
| **Selective attention (assessed with: Stroop (% errors))** | | | | | | | | | | | | |
| 5 | randomised trials | very serious^i^ | not serious^b^ | serious^h^ | serious^c^ | none | 191 | 205 | - | SMD **0 SD**  (0.26 lower to 0.26 higher) | ⨁◯◯◯ Very low^b,c,h,i^ | IMPORTANT |
| **Semantic Fluency (assessed with: Animals (categories))** | | | | | | | | | | | | |
| 7 | randomised trials | serious^g^ | serious^b^ | not serious | not serious | none | 709 | 694 | - | SMD **0.09 SD higher** (0.08 lower to 0.26 higher) | ⨁⨁◯◯ Low^b,g^ | IMPORTANT |
| **Set Shifting (assessed with: TMT B, s)** | | | | | | | | | | | | |
| 13 | randomised trials | serious^g^ | serious^b^ | serious | serious^f^ | none | 796 | 810 | - | SMD **0.14 SD higher** (0.01 lower to 0.3 higher) | ⨁◯◯◯ Very low^b,f,g^ | IMPORTANT |
| **Short term Memory (assessed with: Digit Span Forward)** | | | | | | | | | | | | |
| 11 | randomised trials | very serious^i^ | serious^b^ | serious^h^ | serious^c^ | none | 617 | 678 | - | SMD **0.02 SD higher** (0.16 lower to 0.2 higher) | ⨁◯◯◯ Very low^b,c,h,i^ | IMPORTANT |
| **Working Memory (assessed with: N- Back, Digit Span test backward)** | | | | | | | | | | | | |
| 8 | randomised trials | very serious^i^ | not serious | serious^h^ | serious^c^ | none | 537 | 536 | - | SMD **0.08 SD higher** (0.04 lower to 0.2 higher) | ⨁◯◯◯ Very low^c,h,i^ | IMPORTANT |

**CI:** confidence interval; **SMD:** standardised mean difference

**Explanations**

a. Downgraded one level for risk of bias as the risk-of-bias assessment showed a mixed distribution, with a considerable proportion of evidence rated as some concerns and a non-negligible proportion at high risk across key domains. These limitations may have influenced the estimated effects and reduce overall confidence in the results.

b. Downgraded one level for inconsistency due to variability in the direction and magnitude of effects across included studies, with results not consistently favouring the same intervention effect.

c. Downgraded one level for imprecision as the confidence interval crossed the line of no effect and the total sample size was limited, reducing confidence in the precision of the estimate.

d. Publication bias was suspected due to funnel plot asymmetry suggestive of small-study effects, with smaller studies tending to report larger beneficial effects.

e. Downgraded one level for risk of bias because a substantial proportion of included studies were judged as having some concerns and a notable proportion showed high risk in at least one domain, which may have influenced the estimated effects.

f. Downgraded one level for imprecision because the confidence interval included no effect and the total sample size was limited, resulting in uncertainty about the true magnitude of the effect.

g. Downgraded one level for risk of bias due to methodological limitations across several included trials, including some concerns and high risk in key domains, which may have influenced the estimated effects.

h. Downgraded one level for indirectness because the studies contributing to this outcome did not estimate a fully homogeneous comparison. Some trials compared the intervention with passive or waiting-list controls, whereas others used active comparators, such as exercise alone or cognitive training alone. Therefore, the pooled estimate may reflect partly different clinical questions, including both the overall effect of exercise-based interventions versus no/minimal intervention and the additional effect of combined training over an active intervention. In addition, long-term memory was assessed using different neuropsychological tests across studies, which may capture partially different aspects of memory performance.

i. Downgraded two levels for risk of bias due to a high proportion of studies rated at high risk in key domains, raising serious concerns about the validity of the effect estimates.

# Table 6 Relationship between conventional physical dose and cognitive–motor demand

Values are based on exploratory multilevel meta-regression models using oriented Hedges’ g values, where positive estimates favour exercise. Physical dose refers primarily to weekly exercise volume and intervention duration. Cognitive–motor demand was coded retrospectively at intervention-arm level from published descriptions; therefore, all demand-related analyses should be considered exploratory and hypothesis-generating.

| 6.1. Spearman correlations | | | | | |
| --- | --- | --- | --- | --- | --- |
| **Physical-dose indicator** | **Total cognitive–motor demand** | **Motor complexity** | **Implicit cognitive demand** | **Explicit cognitive training** | **Interpretation** |
| Weekly exercise volume (min/week) | 0.344 | 0.410 | 0.445 | -0.221 | low–moderate |
| Total intervention minutes | 0.200 | 0.253 | 0.241 | -0.110 | low |
| Number of sessions | -0.050 | -0.046 | -0.061 | 0.023 | negligible |
| Session duration (min) | 0.355 | 0.493 | 0.523 | -0.353 | low–moderate |
| Frequency (sessions/week) | 0.067 | 0.041 | 0.067 | 0.009 | negligible |
| Intervention duration (weeks) | -0.126 | -0.049 | -0.082 | -0.096 | low |

| 6.2 Cross-classification of effect sizes by physical dose and cognitive–motor demand | | | | |
| --- | --- | --- | --- | --- |
| Physical-dose group | Cognitive–motor demand group | Effect sizes (k) | Within-dose percentage | Interpretation |
| High physical dose | High cognitive-motor demand | 82.0 | 70.7 | Most high-dose effects were also high demand, but a sizeable minority were low demand. |
| High physical dose | Low cognitive-motor demand | 34.0 | 29.3 |  |
| Low physical dose | High cognitive-motor demand | 23.0 | 45.1 | Low physical dose included both high- and low-demand interventions. |
| Low physical dose | Low cognitive-motor demand | 28.0 | 54.9 |  |

| 6.3 Model fit: physical dose vs cognitive–motor demand | | | | | |
| --- | --- | --- | --- | --- | --- |
| Model | AIC | BIC | logLik | Στ² | Summary |
| Outcome only | 82.140 | 121.780 | -28.070 | 0.107 | Reference model adjusted for cognitive domain. |
| Physical dose | 81.470 | 127.030 | -25.740 | 0.102 | Weekly volume + intervention duration. |
| Cognitive-motor score | 81.950 | 124.560 | -26.980 | 0.107 | Total cognitive–motor demand score. |
| Physical dose + total score | 82.160 | 130.650 | -25.080 | 0.105 | Tests whether total demand adds to physical dose. |
| Physical dose + components | 84.170 | 138.480 | -24.080 | 0.106 | Physical dose plus separate demand components. |

| 6.4 Incremental value of cognitive–motor demand beyond physical dose | | | | | | |
| --- | --- | --- | --- | --- | --- | --- |
| Analysis | Moderator | β | SE | df | p | Interpretation |
| Total demand only | Total cognitive–motor demand score | 0.054 | 0.030 | 11.710 | 0.104 | Positive but not statistically robust. |
| Added to physical dose | Total cognitive–motor demand score | 0.042 | 0.032 | 10.440 | 0.222 | Did not explain additional variability beyond weekly volume and duration. |
| Residual demand only | Residualized demand score | 0.047 | 0.030 | 9.540 | 0.151 | Residual demand not significantly associated with effects. |
| Residual demand added to physical dose | Residualized demand score | 0.047 | 0.030 | 9.770 | 0.146 | No clear incremental value beyond physical dose. |
| Interaction | Weekly volume × total demand | -0.022 | 0.044 | 8.360 | 0.626 | No evidence that dose-response differed by demand level. |
| **Informative contrasts** | **Total cognitive–motor demand score** | **0.023** | **0.042** | **2.490** | **0.629** | **No clear association in study–outcome cells with different demand levels.** |
| Explicit cognitive training contrast | Cog yes vs no | 0.007 | 0.221 | 1.370 | 0.980 | No clear advantage, but this subset was very small. |

| 6.5 Exploratory set-shifting models | | | | | |  |
| --- | --- | --- | --- | --- | --- | --- |
| Model | AIC | BIC | logLik | Στ² | Comment |  |
| **set shifting: physical dose** | **19.090** | **23.260** | **-4.550** | **0.025** | **Reference physical-dose model.** |  |
| set shifting: total complexity | 20.440 | 24.000 | -6.220 | 0.052 | Total score alone did not clearly improve fit. |  |
| set shifting: dose + total complexity | 20.760 | 25.400 | -4.380 | 0.027 | Total score added to physical dose. |  |
| set shifting: dose + components | 22.670 | 27.790 | -3.340 | 0.012 | Lowest residual heterogeneity, but more complex model. |  |
|  |  |  |  |  |  |  |
| Set-shifting component-level coefficients | | | | | | |
| Moderator | β | SE | df | p | Direction | Interpretation |
| Motor complexity | -0.696 | 0.039 | 1.620 | 0.008 | Negative | Exploratory negative association. |
| Implicit cognitive demand | 0.779 | 0.055 | 2.310 | 0.003 | Positive | Exploratory positive association. |
| Explicit cognitive training | -0.022 | 0.024 | 4.760 | 0.402 | Null | No clear association. |
|  |  |  |  |  |  |  |

# Outcome 1: Attention

## 1.1 Results from meta-analysis

A random-effects meta-analysis including 9 effect sizes from 5 studies showed no significant effect on attention (Hedges’ g = −0.08, 95% CI −0.33 to 0.18, p = 0.543). Between-study heterogeneity was moderate to substantial (I² = 66.7%), suggesting relevant variability in the magnitude and direction of effects across studies.

Exploratory dose-response analyses did not show evidence that intervention dose moderated the effect on attention. In the linear dose model, neither weekly exercise volume (β = 0.30, CR2 p = 0.542) nor intervention duration in weeks (β = 0.17, CR2 p = 0.692) was significantly associated with the effect size. Non-linear spline models were considered exploratory and unstable because of the small number of available effect sizes.

Exploratory complexity analyses did not indicate a clear moderating role of cognitive–motor complexity. The revised total complexity score was not associated with attention effects (β = −0.02, CR2 p = 0.581), and motor complexity was also not significant (β = −0.04, CR2 p = 0.619). The explicit cognitive training score showed only a weak, non-robust trend in the CR2 model (β = −0.07, CR2 p = 0.100), but this did not survive correction and should not be interpreted as evidence of moderation. The categorical complexity model also did not show significant differences across complexity classes. The cognitive yes/no model could not be estimated because of insufficient distribution across categories.

## 1.2 Forest Plot

## 1. 3 Funnel Plot

## 1.4 Asymmetry test

Visual inspection of the funnel plot did not suggest marked asymmetry. Egger’s regression test was not significant (p = 0.616), providing no statistical evidence of small-study effects.

## 1.5 Risk of Bias

As percentage (intention-to-treat)

|  | Low risk | Some concerns | High risk |
| --- | --- | --- | --- |
| Randomization process | 61.5 | 38.5 | 0 |
| Deviations from intended interventions | 23.1 | 46.2 | 30.8 |
| Mising outcome data | 61.5 | 0 | 38.5 |
| Measurement of the outcome | 92.3 | 0 | 7.7 |
| Selection of the reported result | 38.5 | 61.5 | 0 |
| Overall Bias | 23.1 | 30.8 | 46.2 |

## 1.6 Leave one out plot

## 1.7 Forest Plot with sub-group sport categories

## 1.8 Sport effect estimates table

| Group | Estimate | CI lower | CI upper |
| --- | --- | --- | --- |
| AEROBIC CONTINUOUS | 0.39 | -0.56 | 1.34 |
| MIND-BODY FLEXIBILITY | -0.47 | -1.78 | 0.83 |
| MIND-BODY SKILL | 0.01 | -0.95 | 0.97 |
| MULTICOMPONENT | 0.15 | -1.02 | 1.31 |
| RESISTANCE TRAINING | 0.21 | -0.95 | 1.38 |

# Outcome 2: Executive Functions

## 2.1 Results from meta-analysis

A random-effects meta-analysis including 12 effect sizes from 6 studies showed a small borderline positive effect on executive functions (Hedges’ g = 0.16, 95% CI −0.00 to 0.33, p = 0.050). Between-study heterogeneity was negligible (I² = 0.0%), indicating that the observed effects were highly consistent across studies.

Exploratory dose-response analyses did not provide clear evidence that dose moderated the intervention effect. Weekly exercise volume was not associated with the effect size (β = 0.01, CR2 p = 0.890). Intervention duration showed a positive but non-significant association (β = 0.16, CR2 p = 0.248). The spline model suggested possible non-linear patterns, but these analyses should be interpreted cautiously because of the limited number of studies and the exploratory nature of the model.

Complexity analyses did not support a moderating role of cognitive–motor complexity for executive functions. The revised total complexity score was not associated with the effect size (β = 0.01, CR2 p = 0.741). Neither motor complexity (β = −0.01, CR2 p = 0.933) nor implicit cognitive enrichment (β = 0.03, CR2 p = 0.890) significantly moderated the results. Models involving explicit cognitive training or cognitive yes/no categorization could not be meaningfully estimated because of insufficient variation in the available data.

## 2.2 Forest Plot

## 2.3 Funnel Plot

## 2.4 Asymmetry test

The funnel plot did not show clear evidence of asymmetry. Egger’s regression test was not significant (p = 0.271), and trim-and-fill did not impute missing studies, suggesting no evidence of small-study effects.

## 2.5 Risk of Bias

As percentage (intention-to-treat)

|  | Low risk | Some concerns | High risk |
| --- | --- | --- | --- |
| Randomization process | 61.5 | 38.5 | 0 |
| Deviations from intended interventions | 23.1 | 46.2 | 30.8 |
| Mising outcome data | 61.5 | 0 | 38.5 |
| Measurement of the outcome | 92.3 | 0 | 7.7 |
| Selection of the reported result | 38.5 | 61.5 | 0 |
| Overall Bias | 23.1 | 30.8 | 46.2 |

## 2.6 Leave one out plot

## 2.7 Forest Plot with sub-group sport categories

## 2.8 Sport effect estimates table

| Group | Estimate | CI lower | CI upper |
| --- | --- | --- | --- |
| AEROBIC CONTINUOUS | -0.17 | -0.40 | 0.06 |
| INTERVAL HIGH INTENSITY | -0.18 | -0.47 | 0.11 |
| MIND-BODY SKILL | -0.03 | -0.81 | 0.75 |
| RESISTANCE TRAINING | -0.12 | -0.75 | 0.50 |

# Outcome 3: Global Cognitive Functioning

## 3.1 Results from meta-analysis

A random-effects meta-analysis including 30 effect sizes from 19 studies showed a significant positive effect on global cognition (Hedges’ g = 0.55, 95% CI 0.38 to 0.73, p < 0.001). However, between-study heterogeneity was substantial (I² = 75.6%), indicating considerable variability in the magnitude of effects across studies.

Exploratory dose-response analyses did not show evidence that intervention dose explained this variability. Weekly exercise volume was not significantly associated with the effect size (β = 0.05, CR2 p = 0.705), nor was intervention duration in weeks (β = −0.06, CR2 p = 0.551). Non-linear spline models were interpretable with caution but did not provide robust evidence of dose-dependent effects.

Complexity analyses did not show consistent evidence that global cognition effects varied according to the overall cognitive–motor complexity of the intervention. The revised total complexity score was not significant (β = 0.02, CR2 p = 0.539). Motor complexity (β = −0.12, CR2 p = 0.608) and implicit cognitive enrichment (β = 0.13, CR2 p = 0.582) were also not significant. The explicit cognitive training score showed a positive association in the uncorrected CR2 model (β = 0.07, CR2 p = 0.018), suggesting that interventions with a more explicit cognitive-training component may have larger effects on global cognition. However, this association did not remain significant after FDR correction and should therefore be interpreted as exploratory. The categorical complexity model did not indicate significant differences across complexity classes. The cognitive yes/no model could not be fitted because too few effect sizes were available in the cognitive-added category.3.1 Forest Plot

## 3.2 Forest Plot

## 3.3 Funnel plot

## 3.4 Asymmetry test

Funnel plot inspection suggested possible asymmetry. Egger’s regression test was significant (p < 0.001), indicating potential small-study effects. Trim-and-fill imputed five potentially missing studies and yielded an attenuated but still positive adjusted estimate (g = 0.43, 95% CI 0.23 to 0.63), suggesting that the effect may be smaller than the unadjusted estimate but remains in favor of the intervention.

## 3.5 Risk of Bias

As percentage (intention-to-treat)

|  | Low risk | Some concerns | High risk |
| --- | --- | --- | --- |
| Randomization process | 61.5 | 38.5 | 0 |
| Deviations from intended interventions | 23.1 | 46.2 | 30.8 |
| Mising outcome data | 61.5 | 0 | 38.5 |
| Measurement of the outcome | 92.3 | 0 | 7.7 |
| Selection of the reported result | 38.5 | 61.5 | 0 |
| Overall Bias | 23.1 | 30.8 | 46.2 |

## 3.6 Leave one out plot

## 3.7 Forest Plot with sub-group sport categories

## 3.8 Sport effect estimates table

| Group | Estimate | CI lower | CI upper |
| --- | --- | --- | --- |
| AEROBIC CONTINUOUS | 0.56 | 0.23 | 0.89 |
| INTERVAL HIGH INTENSITY | 0.44 | -0.10 | 0.97 |
| MIND-BODY SKILL | 0.54 | 0.19 | 0.90 |
| MULTICOMPONENT COMBINED | 0.65 | 0.31 | 1.00 |
| RESISTANCE TRAINING | 0.65 | 0.32 | 0.99 |
| STRENGHT TRAINING | 0.25 | -0.29 | 0.79 |

# Outcome 4: Long Term Memory

## 4.1 Results from meta-analysis

A random-effects meta-analysis including 11 effect sizes from 6 studies showed no significant effect on long-term memory (Hedges’ g = 0.11, 95% CI −0.06 to 0.27, p = 0.205). Between-study heterogeneity was low to moderate (I² = 36.3%), suggesting limited inconsistency across studies.

Exploratory dose-response analyses did not indicate that intervention dose moderated long-term memory outcomes. Weekly exercise volume was not significantly associated with the effect size (β = 0.25, CR2 p = 0.333), and intervention duration also showed no association (β = 0.05, CR2 p = 0.823). Non-linear spline analyses were considered exploratory and unstable because of the limited number of effect sizes.

Complexity models did not provide evidence that cognitive–motor complexity moderated long-term memory effects. The revised total complexity score was not significant (β = −0.06, CR2 p = 0.218). Motor complexity showed a negative but non-significant association (β = −0.15, CR2 p = 0.246), whereas implicit cognitive enrichment was not significant (β = 0.04, CR2 p = 0.661). Explicit cognitive training was also not associated with the effect size (β = 0.02, CR2 p = 0.686). The categorical complexity model did not indicate significant differences across complexity classes, and the cognitive yes/no model could not be estimated due to insufficient data.

## 4.2 Forest Plot

## 4.3 Funnel Plot

## 4.4 Asymmetry test

The funnel plot appeared broadly symmetrical. Egger’s regression test was not significant (p = 0.680), providing no statistical evidence of small-study effects. Trim-and-fill imputed three studies and produced a slightly larger adjusted estimate (g = 0.19, 95% CI 0.02 to 0.35), but this should be interpreted cautiously given the non-significant Egger test and the small number of studies.

## 4.5 Risk of Bias

As percentage (intention-to-treat)

|  | Low risk | Some concerns | High risk |
| --- | --- | --- | --- |
| Randomization process | 72.7 | 27.3 | 0 |
| Deviations from intended interventions | 45.5 | 18.2 | 36.4 |
| Mising outcome data | 90.9 | 0 | 9.1 |
| Measurement of the outcome | 90.9 | 0 | 9.1 |
| Selection of the reported result | 72.7 | 18.2 | 9.1 |
| Overall Bias | 45.5 | 18.2 | 36.4 |

## 4.6 Leave one out plot

## 4.7 Forest Plot with sub-group sport categories

## 4.8 Sport effect estimates table

| Group | Estimate | CI lower | CI upper |
| --- | --- | --- | --- |
| AEROBIC CONTINUOUS | 0.08 | -0.54 | 0.70 |
| INTERVAL HIGH INTENSITY | 0.00 | -0.87 | 0.88 |
| MIND-BODY FLEXIBILITY | -0.07 | -1.03 | 0.90 |
| MIND-BODY SKILL | 0.45 | -0.22 | 1.13 |
| MULTICOMPONENT COMBINED | -0.13 | -0.89 | 0.64 |
| RESISTANCE TRAINING | -0.11 | -0.88 | 0.65 |

# Outcome 5: Phonemic Fluency

## 5.1 Results from meta-analysis

A random-effects meta-analysis including 14 effect sizes from 8 studies showed a small, non-significant effect on phonemic fluency (Hedges’ g = 0.15, 95% CI −0.02 to 0.31, p = 0.080). Between-study heterogeneity was moderate (I² = 54.9%), indicating variability in the observed effects across studies.

Exploratory dose-response analyses did not provide robust evidence of moderation by intervention dose. Weekly exercise volume showed a positive but non-significant association with the effect size (β = 0.18, CR2 p = 0.114), whereas intervention duration was not associated with the effect (β = −0.03, CR2 p = 0.769). Spline analyses did not provide robust evidence of non-linear dose effects and should be interpreted cautiously.

Complexity analyses suggested possible but non-robust associations with specific components of cognitive–motor complexity. The revised total complexity score was not significant (β = −0.03, CR2 p = 0.319). Motor complexity was negatively associated with the effect size in the uncorrected CR2 model (β = −0.48, CR2 p = 0.039), while implicit cognitive enrichment showed a positive association (β = 0.42, CR2 p = 0.046). However, these associations did not remain significant after FDR correction, so they should be interpreted as exploratory signals rather than confirmatory evidence. Explicit cognitive training was not significantly associated with the effect (β = 0.04, CR2 p = 0.145), and the categorical complexity model did not show significant differences across classes. The cognitive yes/no model could not be estimated because of insufficient distribution across categories.

## 5.2 Forest Plot

## 5.3 Funnel Plot

## 5.4 Asymmetry test

Visual inspection of the funnel plot did not suggest relevant asymmetry. Egger’s regression test was not significant (p = 0.871), indicating no statistical evidence of small-study effects. Trim-and-fill imputed two studies and yielded an attenuated adjusted estimate (g = 0.07, 95% CI −0.11 to 0.25), consistent with the non-significant primary finding.

## 5.5 Risk of Bias

As percentage (intention-to-treat)

|  | Low risk | Some concerns | High risk |
| --- | --- | --- | --- |
| Randomization process | 61.5 | 38.5 | 0 |
| Deviations from intended interventions | 23.1 | 46.2 | 30.8 |
| Mising outcome data | 61.5 | 0 | 38.5 |
| Measurement of the outcome | 92.3 | 0 | 7.7 |
| Selection of the reported result | 38.5 | 61.5 | 0 |
| Overall Bias | 23.1 | 30.8 | 46.2 |

## 5.6 Leave one out plot

## 5.7 Forest Plot with sub-group sport categories

## 5.8 Sport effect estimates table

| Group | Estimate | CI lower | CI upper |
| --- | --- | --- | --- |
| AEROBIC CONTINUOUS | 0.12 | -0.18 | 0.42 |
| INTERVAL HIGH INTENSITY | -0.43 | -1.05 | 0.18 |
| MIND-BODY FLEXIBILITY | 0.26 | -0.25 | 0.78 |
| MIND-BODY SKILL | 0.25 | -0.22 | 0.71 |
| MULTICOMPONENT COMBINED | 0.14 | -0.26 | 0.55 |
| RESISTANCE TRAINING | 0.31 | -0.05 | 0.66 |
| STRENGHT TRAINING | 0.15 | -0.39 | 0.69 |

# Outcome 6: Processing Speed

## 6.1 Results from meta-analysis

A random-effects meta-analysis including 18 effect sizes from 11 studies showed no significant effect on processing speed (Hedges’ g = 0.06, 95% CI −0.11 to 0.23, p = 0.500). Between-study heterogeneity was moderate (I² = 55.2%), suggesting variability in effect estimates across studies.

Exploratory dose-response analyses did not show statistically significant moderation by dose. Weekly exercise volume showed a positive but non-significant association (β = 0.13, CR2 p = 0.104), while intervention duration showed a negative but non-significant association (β = −0.14, CR2 p = 0.096). These trends did not survive correction and should be interpreted cautiously. Non-linear spline models did not provide robust evidence of dose-response effects.

Complexity analyses did not indicate a moderating role of cognitive–motor complexity for processing speed. The revised total complexity score was not significant (β = 0.02, CR2 p = 0.357), and neither motor complexity (β = 0.03, CR2 p = 0.827) nor implicit cognitive enrichment (β = 0.04, CR2 p = 0.788) showed significant associations. Explicit cognitive training was also not associated with the effect (β = −0.09, CR2 p = 0.610). The categorical complexity model did not show differences across intervention complexity classes, and the cognitive yes/no model could not be fitted because of insufficient data.

## 6.2 Forest Plot

## 6.3 Funnel Plot

## 6.4 Asymmetry test

The funnel plot did not indicate substantial asymmetry. Egger’s regression test was not significant (p = 0.845), providing no statistical evidence of small-study effects. Trim-and-fill imputed two studies and yielded an adjusted estimate close to zero (g = 0.01, 95% CI −0.16 to 0.18), consistent with the null primary result.

## 6.5 Risk of Bias

As percentage (intention-to-treat)

|  | Low risk | Some concerns | High risk |
| --- | --- | --- | --- |
| Randomization process | 61.5 | 38.5 | 0 |
| Deviations from intended interventions | 23.1 | 46.2 | 30.8 |
| Mising outcome data | 61.5 | 0 | 38.5 |
| Measurement of the outcome | 92.3 | 0 | 7.7 |
| Selection of the reported result | 38.5 | 61.5 | 0 |
| Overall Bias | 23.1 | 30.8 | 46.2 |

## 6.6 Leave one out plot

## 6.7 Forest Plot with sub-group sport categories

## 6.8 Sport effect estimates table

| Group | Estimate | CI lower | CI upper |
| --- | --- | --- | --- |
| AEROBIC CONTINUOUS | 0.25 | -0.09 | 0.58 |
| INTERVAL HIGH INTENSITY | 0.34 | -0.30 | 0.97 |
| MIND-BODY FLEXIBILITY | -0.29 | -1.05 | 0.47 |
| MIND-BODY SKILL | -0.25 | -0.55 | 0.05 |
| MULTICOMPONENT COMBINED | 0.01 | -0.48 | 0.49 |
| RESISTANCE TRAINING | -0.25 | -0.62 | 0.12 |
| STRENGHT TRAINING | -0.29 | -0.78 | 0.19 |

# Outcome 7: Selective Attention

## 7.1 Results from meta-analysis

A random-effects meta-analysis including 9 effect sizes from 5 studies showed no effect on selective attention (Hedges’ g = −0.00, 95% CI −0.27 to 0.26, p = 0.979). Between-study heterogeneity was low to moderate (I² = 37.4%), indicating relatively limited variability across studies.

Exploratory dose-response analyses did not show evidence of moderation by intervention dose. Weekly exercise volume was not significantly associated with the effect size (β = −0.23, CR2 p = 0.277), nor was intervention duration (β = 0.16, CR2 p = 0.310). Spline models were not considered interpretable because of the small number of available effect sizes.

Complexity analyses provided limited and non-robust evidence of moderation. The revised total complexity score was positively associated with selective attention effects in the uncorrected CR2 model (β = 0.05, CR2 p = 0.046), but this association did not remain significant after FDR correction. Motor complexity (β = 0.47, CR2 p = 0.598), implicit cognitive enrichment (β = −0.32, CR2 p = 0.673), and explicit cognitive training (β = 0.05, CR2 p = 0.601) were not significant. The categorical complexity model did not provide robust evidence of differences between complexity classes. The cognitive yes/no model could not be estimated due to insufficient category distribution.

## 7.2 Forest Plot

## 7.3 Funnel Plot

## 7.4 Asymmetry test

Visual inspection of the funnel plot did not suggest clear asymmetry. Egger’s regression test was not significant (p = 0.795), indicating no statistical evidence of small-study effects.

## 7.5 Risk of Bias

As percentage (intention-to-treat)

|  | Low risk | Some concerns | High risk |
| --- | --- | --- | --- |
| Randomization process | 61.5 | 38.5 | 0 |
| Deviations from intended interventions | 23.1 | 46.2 | 30.8 |
| Mising outcome data | 61.5 | 0 | 38.5 |
| Measurement of the outcome | 92.3 | 0 | 7.7 |
| Selection of the reported result | 38.5 | 61.5 | 0 |
| Overall Bias | 23.1 | 30.8 | 46.2 |

## 7.6 Leave one out plot

## 7.7 Forest Plot with sub-group sport categories

## 7.8 Sport effect estimates table

| Group | Estimate | CI lower | CI upper |
| --- | --- | --- | --- |
| AEROBIC CONTINUOUS | -0.43 | -1.06 | 0.19 |
| INTERVAL HIGH INTENSITY | 0.15 | -0.48 | 0.79 |
| MIND-BODY SKILL | 0.18 | -0.51 | 0.88 |
| RESISTANCE TRAINING | -0.15 | -0.75 | 0.46 |

# Outcome 8: Semantic Fluency (secondary analysis)

## 8.1 Results from meta-analysis

A random-effects meta-analysis including 14 effect sizes from 7 studies showed no significant effect on semantic fluency (Hedges’ g = 0.09, 95% CI −0.08 to 0.26, p = 0.289). Between-study heterogeneity was moderate (I² = 57.0%), suggesting variability in the magnitude of effects across studies.

Exploratory dose-response analyses did not provide robust evidence that intervention dose moderated semantic fluency outcomes. Weekly exercise volume was not significant (β = 0.04, CR2 p = 0.555), and intervention duration showed a negative but non-significant association (β = −0.21, CR2 p = 0.117). Spline models did not provide evidence of non-linear dose effects.

Complexity analyses suggested possible exploratory associations with specific complexity components. The revised total complexity score was not associated with semantic fluency effects (β = 0.01, CR2 p = 0.546). Motor complexity showed a negative association in the uncorrected CR2 model (β = −0.30, CR2 p = 0.049), while implicit cognitive enrichment showed a positive association (β = 0.33, CR2 p = 0.018). However, these associations did not survive FDR correction and should therefore be interpreted cautiously. Explicit cognitive training could not be meaningfully interpreted for this outcome, and the categorical complexity model did not show significant differences across classes. The cognitive yes/no model was not fitted because of insufficient data.

## 8.2 Forest Plot

## 8.3 Funnel Plot

## 8.4 Asymmetry test

The funnel plot did not provide strong evidence of asymmetry. Egger’s regression test was not significant (p = 0.130), suggesting no clear evidence of small-study effects. Trim-and-fill did not impute missing studies, and the adjusted estimate was unchanged.

## 8.5 Risk of Bias

As percentage (intention-to-treat)

|  | Low risk | Some concerns | High risk |
| --- | --- | --- | --- |
| Randomization process | 61.5 | 38.5 | 0 |
| Deviations from intended interventions | 23.1 | 46.2 | 30.8 |
| Mising outcome data | 61.5 | 0 | 38.5 |
| Measurement of the outcome | 92.3 | 0 | 7.7 |
| Selection of the reported result | 38.5 | 61.5 | 0 |
| Overall Bias | 23.1 | 30.8 | 46.2 |

## 8.6 Leave one out plot

## 8.7 Forest Plot with sub-group sport categories

## 8.8 Sport effect estimates table

| Group | Estimate | CI lower | CI upper |
| --- | --- | --- | --- |
| AEROBIC CONTINUOUS | 0.10 | -0.21 | 0.40 |
| INTERVAL HIGH INTENSITY | -0.22 | -0.85 | 0.41 |
| MIND-BODY FLEXIBILITY | 0.24 | -0.30 | 0.77 |
| MIND-BODY SKILL | 0.09 | -0.42 | 0.59 |
| MULTICOMPONENT COMBINED | 0.20 | -0.16 | 0.56 |
| RESISTANCE TRAINING | 0.26 | -0.10 | 0.62 |
| STRENGTH TRAINING | 0.16 | -0.41 | 0.72 |

# Outcome 9: Set Shifting

## 9.1 Results from meta-analysis

A random-effects meta-analysis including 20 effect sizes from 13 studies showed a small, non-significant effect on set shifting (Hedges’ g = 0.14, 95% CI −0.02 to 0.30, p = 0.077). Between-study heterogeneity was moderate (I² = 54.2%), indicating some inconsistency in effect estimates across studies.

Exploratory dose-response analyses did not show statistically robust moderation by dose. Weekly exercise volume showed a positive but non-significant association (β = 0.14, CR2 p = 0.094), and intervention duration also showed no significant association (β = −0.09, CR2 p = 0.182). Non-linear spline models did not provide robust evidence of dose-dependent effects.

Complexity analyses suggested that specific components of intervention complexity may be relevant for set shifting. The revised total complexity score was not significantly associated with the effect size (β = 0.01, CR2 p = 0.439). However, motor complexity showed a significant negative association (β = −0.52, CR2 p < 0.001), whereas implicit cognitive enrichment showed a significant positive association (β = 0.54, CR2 p < 0.001). Both associations remained significant after FDR correction, suggesting that the structure of the intervention may differentially relate to set-shifting outcomes. Specifically, interventions with greater implicit cognitive enrichment appeared to be associated with more favorable effects, whereas higher motor complexity, when modeled separately, was associated with less favorable effects. Explicit cognitive training was not significant (β = 0.02, CR2 p = 0.577), and the categorical complexity model did not show significant differences across classes. The cognitive yes/no model could not be estimated because of insufficient data.

## 9.2 Forest Plot

## 9.3 Funnel Plot

## 9.4 Asymmetry test

Visual inspection of the funnel plot did not suggest marked asymmetry. Egger’s regression test was not significant (p = 0.765), providing no statistical evidence of small-study effects. Trim-and-fill imputed three studies and yielded a larger adjusted estimate (g = 0.22, 95% CI 0.06 to 0.39), but this should be interpreted cautiously because the primary Egger test was not significant.

## 9.5 Risk of Bias

As percentage (intention-to-treat)

|  | Low risk | Some concerns | High risk |
| --- | --- | --- | --- |
| Randomization process | 61.5 | 38.5 | 0 |
| Deviations from intended interventions | 23.1 | 46.2 | 30.8 |
| Mising outcome data | 61.5 | 0 | 38.5 |
| Measurement of the outcome | 92.3 | 0 | 7.7 |
| Selection of the reported result | 38.5 | 61.5 | 0 |
| Overall Bias | 23.1 | 30.8 | 46.2 |

## 9.6 Leave one out plot

## 9.7 Forest Plot with sub-group sport categories

## 9.8 Sport effect estimates table

| Group | Estimate | CI lower | CI upper |
| --- | --- | --- | --- |
| AEROBIC CONTINUOUS | -0.10 | -0.41 | 0.22 |
| INTERVAL HIGH INTENSITY | 0.42 | -0.25 | 1.10 |
| MIND-BODY FLEXIBILITY | 0.28 | -0.51 | 1.07 |
| MIND-BODY SKILL | -0.35 | -0.67 | -0.03 |
| MULTICOMPONENT COMBINED | -0.19 | -0.68 | 0.30 |
| RESISTANCE TRAINING | -0.31 | -0.63 | 0.02 |
| STRENGTH TRAINING | -0.02 | -0.62 | 0.58 |

# Outcome 10: Short-Term Memory (secondary analysis)

## 10.1 Results from meta-analysis

A random-effects meta-analysis including 17 effect sizes from 11 studies showed no significant effect on short-term memory (Hedges’ g = 0.02, 95% CI −0.16 to 0.20, p = 0.814). Between-study heterogeneity was moderate (I² = 58.1%), suggesting variability in effects across studies.

Exploratory dose-response analyses did not provide evidence that dose moderated short-term memory effects. Weekly exercise volume was not significantly associated with the effect size (β = 0.17, CR2 p = 0.208), and intervention duration was also not significant (β = −0.11, CR2 p = 0.374). Spline models did not provide robust evidence of non-linear dose-response patterns.

Complexity analyses did not indicate a clear moderating role of cognitive–motor complexity. The revised total complexity score was not significant (β = −0.02, CR2 p = 0.503). Motor complexity (β = 0.14, CR2 p = 0.313), implicit cognitive enrichment (β = −0.16, CR2 p = 0.316), and explicit cognitive training (β = −0.08, CR2 p = 0.485) were also not significant. The categorical complexity model did not indicate significant differences across classes, and the cognitive yes/no model could not be fitted because of insufficient data.

## 10.2 Forest Plot

## 10.3 Funnel Plot

## 10.4 Asymmetry test

The funnel plot appeared broadly symmetrical. Egger’s regression test was not significant (p = 0.915), indicating no evidence of small-study effects or publication bias. Trim-and-fill did not impute missing studies, and the adjusted estimate remained unchanged.

## 10.5 Risk of Bias

As percentage (intention-to-treat)

|  | Low risk | Some concerns | High risk |
| --- | --- | --- | --- |
| Randomization process | 61.5 | 38.5 | 0 |
| Deviations from intended interventions | 23.1 | 46.2 | 30.8 |
| Mising outcome data | 61.5 | 0 | 38.5 |
| Measurement of the outcome | 92.3 | 0 | 7.7 |
| Selection of the reported result | 38.5 | 61.5 | 0 |
| Overall Bias | 23.1 | 30.8 | 46.2 |

## 10.6 Leave one out plot

## 10.7 Forest Plot with sub-group sport categories

## 10.8 Sport effect estimates table

| Group | Estimate | CI lower | CI upper |
| --- | --- | --- | --- |
| AEROBIC CONTINUOUS | 0.11 | -0.30 | 0.53 |
| INTERVAL HIGH INTENSITY | 0.26 | -0.43 | 0.96 |
| MIND-BODY FLEXIBILITY | -0.24 | -1.07 | 0.59 |
| MIND-BODY SKILL | 0.33 | -0.10 | 0.76 |
| MULTICOMPONENT COMBINED | 0.00 | -0.42 | 0.42 |
| RESISTANCE TRAINING | 0.23 | -0.21 | 0.67 |
| STRENGTH TRAINING | -0.28 | -1.39 | 0.84 |

# Outcome 11: Working Memory

## 11.1 Results from meta-analysis

A random-effects meta-analysis including 13 effect sizes from 8 studies showed no significant effect on working memory (Hedges’ g = 0.08, 95% CI −0.04 to 0.20, p = 0.196). Between-study heterogeneity was negligible (I² = 0.0%), suggesting a high degree of consistency across studies.

Exploratory dose-response analyses did not show evidence that intervention dose moderated working memory effects. Weekly exercise volume was not significant (β = 0.02, CR2 p = 0.615), while intervention duration showed a negative trend that did not reach conventional significance (β = −0.12, CR2 p = 0.078). This trend did not survive correction and should be interpreted cautiously. Spline models did not provide evidence of robust non-linear dose effects.

Complexity analyses did not support a moderating role of cognitive–motor complexity. The revised total complexity score was not associated with the effect size (β = 0.01, CR2 p = 0.615), and motor complexity was also not significant (β = 0.01, CR2 p = 0.704). Implicit cognitive enrichment and explicit cognitive training models were not interpretable or could not be meaningfully estimated for this outcome. The categorical complexity model did not show significant differences across complexity classes. The cognitive yes/no model was not fitted because of insufficient data.

## 11.2 Forest Plot

## 11.3 Funnel Plot

## 11.4 Asymmetry test

Visual inspection of the funnel plot did not suggest relevant asymmetry. Egger’s regression test was not significant (p = 0.392), providing no statistical evidence of small-study effects. Trim-and-fill imputed one study, yielding an adjusted estimate very similar to the original result (g = 0.07, 95% CI −0.05 to 0.19).

## 11.5 Risk of Bias

As percentage (intention-to-treat)

|  | Low risk | Some concerns | High risk |
| --- | --- | --- | --- |
| Randomization process | 61.5 | 38.5 | 0 |
| Deviations from intended interventions | 23.1 | 46.2 | 30.8 |
| Mising outcome data | 61.5 | 0 | 38.5 |
| Measurement of the outcome | 92.3 | 0 | 7.7 |
| Selection of the reported result | 38.5 | 61.5 | 0 |
| Overall Bias | 23.1 | 30.8 | 46.2 |

## 11.6 Leave one out plot

## 11.7 Forest Plot with sub-group sport categories

## 11.8 Sport effect estimates table

| Group | Estimate | CI lower | CI upper |
| --- | --- | --- | --- |
| AEROBIC CONTINUOUS | 0.06 | -0.17 | 0.30 |
| MIND-BODY SKILL | 0.33 | -0.11 | 0.77 |
| MULTICOMPONENT COMBINED | -0.05 | -0.29 | 0.19 |
| RESISTANCE TRAINING | 0.14 | -0.11 | 0.38 |
| STRENGTH TRAINING | -0.13 | -0.97 | 0.71 |
